# Assembled Hyaluronic Acid Therapeutics for Targeting M1 Macrophages and Regulating Immune Homeostasis for Ulcerative Colitis Treatment

**DOI:** 10.1002/advs.77872

**Published:** 2026-09-27

**Authors:** Jianqin Wan, Andi Ma, Yiqiong Zhang, Minghao Zhang, Yifeng Lin, Xiaoling Zhang, Hongjun Li, Weidong Han

**Affiliations:** ^1^ Zhejiang Cancer Hospital Hangzhou Institute of Medicine (HIM) Chinese Academy of Sciences Hangzhou Zhejiang China; ^2^ Liangzhu Laboratory Zhejiang University Medical Center Hangzhou Zhejiang China

**Keywords:** hyaluronic acid‐based prodrug design, intestinal immune homeostasis, M1 macrophage‐targeted therapy, self‐assembled nanoparticles, ulcerative colitis

## Abstract

Hyperactivated M1 macrophages with excessive reactive oxygen species (ROS) exacerbate intestinal inflammation, driving the progression of inflammatory bowel diseases (IBDs), for instance, ulcerative colitis. Current standard treatments for IBDs show limited efficacy and off‐target side effects. Herein, we develop an oral, drug‐free hyaluronic acid (HA)‐based therapeutics that could self‐assemble in aqueous solution, accumulate in inflamed colons and attenuate M1‐like activation, thereby rebalancing the colonic immune landscape. Such drug‐free therapeutics are generated by the conjugation of unsaturated fatty acids to HA via dynamic covalent boronate‐catechol ester bonds. Mechanistically, the nano‐assemblies (termed uhNAs) effectively scavenge ROS, dampen NF‐κB signaling, and diminish pro‐inflammatory cytokines in M1‐like phenotypes. In DSS‐induced acute colitis, oral uhNAs treatment elicits significant decreases in M1‐like macrophages, CD8^+^ T cells, and neutrophils with concomitant increases in Tregs. In chronic and relapsing DSS colitis, uhNAs also confer robust protection, supporting sustained colonic immune remodeling during recurrent intestinal inflammation. Single‐cell RNA‐sequencing corroborates these shifts and maps the myeloid, B and T‐cell programs, highlighting the downregulated inflammatory responses following uhNAs treatment. Notably, uhNAs‐OA outperform physical HA (or HA‐PBA)/oleic‐acid mixtures, and two clinically established IBD drugs (5‐aminosalicylic acid and dexamethasone), protecting mice models against both acute and chronic colitis.

## Graphical abstract

An orally administered, drug‐free hyaluronic acid nanoassembly, uhNAs‐OA, accumulates in the inflamed colon and is preferentially internalized by M1‐like macrophages via HA‐CD44 interactions. uhNAs‐OA scavenges excess ROS, suppresses NF‐κB signaling and remodels the colonic immune landscape, resulting in robust therapeutic efficacy in acute, chronic and relapsing colitis.

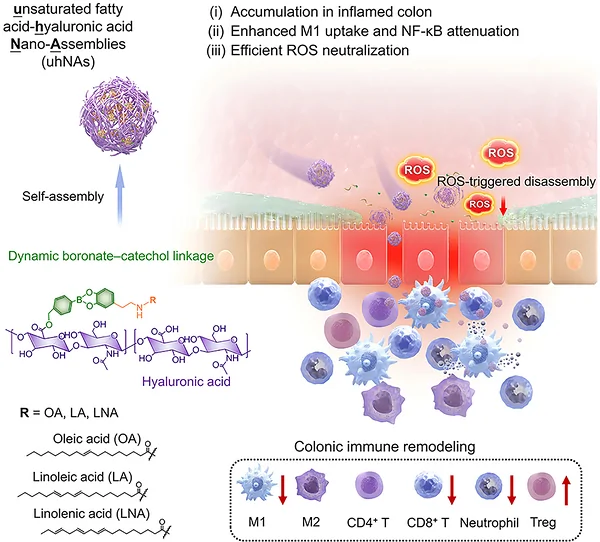

## 1 Introduction

Ulcerative colitis (UC), an inflammatory bowel disease (IBD), poses a significant and growing clinical burden worldwide [1]. Accumulating evidence has demonstrated the crucial role of pro‐inflammatory M1 macrophages in the onset of colitis [2]. Hyperactivated M1 macrophages are able to produce high level of reactive oxygen species (ROS) [3, 4], which exacerbates the damage to colonic epithelium and triggers the activation of NF‐κB, MAPK and JAK‐STAT signalings, driving the release of pro‐inflammatory cytokines like TNF‐α and IL‐6, thereby intensifying the inflammatory response [5, 6]. Current standard therapies for UC mainly include aminosalicylates (e.g., sulfasalazine and mesalamine), corticosteroids, immunomodulators and biologics (e.g., antibodies against pro‐inflammatory cytokines); however, their therapeutic efficacy remains limited, and durable responses are not consistently achieved [7]. Moreover, long‐term use of these small molecules or biologics leads to significant off‐target side effects and complications. Thus, a drug‐free therapeutic strategy that selectively targets pro‐inflammatory M1 macrophages, scavenges excessive ROS, and reprograms the colonic immune microenvironment may represent a promising alternative for UC therapy.

Despite advances in oral drug delivery, therapies for UC still suffer from suboptimal localization and residence at inflamed colon sites. Endowing carriers with mucoadhesion enables prolonged intestinal retention [8, 9, 10, 11], yet safe, cell‐targeted platforms without anti‐inflammatory small molecules or biologics remain limited. Hyaluronic acid (HA), a biocompatible polysaccharide, can engage CD44 receptors, which are abundant on the surface of inflammatory cells, particularly activated M1 macrophages [12, 13]. Free HA or HA‐based nanoformulations have been widely explored for IBD treatments, owing to their potent immunomodulatory activity, notably the modulation of macrophage polarization [14] and the induction of regulatory CD4^+^ T cells (Tregs) [15]. Prior studies have conjugated bioactive molecules (e.g., bilirubin) to HA to achieve selective M1 targeting, thereby enhancing the therapeutic outcomes [16, 17]. Nonetheless, such HA‐derived conjugates are constrained by intrinsically low payloads from one‐site conjugation. Accordingly, there is an urgent need to develop more sophisticated nano‐delivery systems that integrate high drug loading with selective engagement of CD44^+^ macrophages and efficient ROS scavenging. We and other groups [18, 19] previously demonstrated that phenylboronic acid (PBA) modification of carboxyl groups proceeded with high efficiency. Moreover, the PBA moiety not only enables further dynamic covalent linkages with cis‐diols [20, 21], but also possesses robust ROS‐scavenging activity [22, 23]. Thus, incorporating PBA to HA offers significant advantages, alleviating excessive oxidative stress while enabling tunable architectures.

**FIGURE 1:**
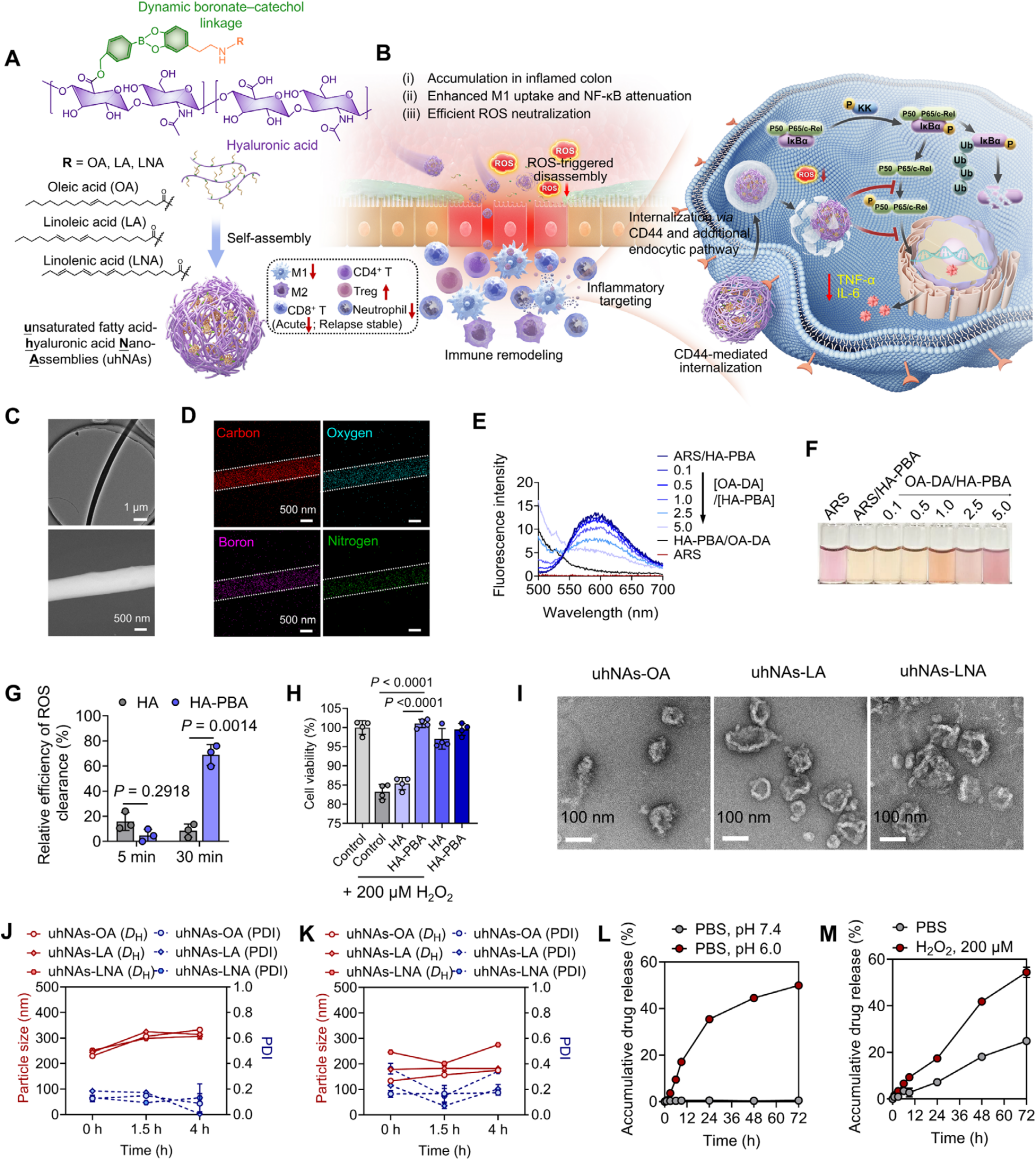
Design and characterization of ROS‐responsive fatty acid‐HA nanoclusters for UC treatment. (A) Chemical structures of fatty acid‐HA conjugates (HA‐OA, HA‐LA, and HA‐LNA) and their self‐assembly into uhNAs. (B) Schematic of the strategy for uhNAs‐enabled immune remodeling in colitis. Following administration, uhNAs accumulated in inflamed colon, scavenged excessive ROS and dampened the inflammatory responses in activated macrophages. (C) Scan electron microscopy (SEM) images of HA‐PBA. (D) Representative EDS elemental mapping of HA‐PBA. Scale bar, 1 µm or 500 nm. (E, F) Competitive fluorescence binding assay. ARS was mixed with HA‐PBA at a molar ratio of 1: 1 in PBS solution (pH 7.4), and then serial molar ratios of OA‐DA (0.1–5.0 equiv. of HA‐PBA) were added into the mixture. The fluorescence signal was recorded by a fluorescence spectrophotometer. (E) Emission spectra of the ARS/HA‐PBA complex upon the incorporation of OA‐DA. Excitation wavelength: 468 nm. (F) Photographs of the corresponding solutions in (E). (G) Relative ROS scavenging efficiency of HA and HA‐PBA, assessed using an AAPH‐induced oxidative stress model in the presence of indocyanine green (ICG). HA and HA‐PBA were used at an HA‐equivalent concentration of 1 mg ml^−^^1^. (H) Viability of Caco2 cells following 24 h treatment with or without 1 mg mL^−1^ HA or HA‐PBA in the presence of H_2_O_2_ (200 µM). I) Representative transmission electron microscopy (TEM) images of uhNAs‐OA, ‐LA, or ‐LNA. Stability of uhNAs in SGF (J) or SIF (K) within 4 h. Particle size and polydisperse index were recorded by DLS analysis. Release kinetics of uhNAs‐OA under acidic conditions (pH 6.0, L) or in the presence of 200 µM H_2_O_2_ (M). Data were presented as the mean ± SD (n = 3 biologically independent samples for G and n = 4 biologically independent samples for H). Statistical significance was examined by one‐way ANOVA with Tukey's multiple comparisons test (H) or two‐way ANOVA with Sidak's multiple comparisons test (G).

Here, we developed a series of dynamic fatty acid‐HA nanoassemblies (uhNAs) for colitis treatment, in which HA promoted accumulation in inflamed colon tissue, PBA endowed the nanoassemblies with intrinsic ROS‐scavenging capacity, and lipid conjugation drove the self‐assembly while potentiating the anti‐inflammatory efficacy (Figure 1A,B). Specifically, PBA was tethered to HA to yield boronated HA conjugates (HA‐PBA), which were further functionalized with monounsaturated (e.g., oleic acid (OA)) or polyunsaturated (e.g., linoleic acid (LA) and linolenic acid (LNA)) fatty acids via dynamic covalent boronate‐catechol ester bonds (Figure 1A). These conjugates spontaneously organized into defined nanoassemblies in aqueous solution and oral administration of these assemblies exhibited significantly greater therapeutic efficacy than physical mixtures of HA (or HA‐PBA) and OA, as well as two clinically used IBD therapeutics, 5‐aminosalicylic acid (5‐ASA) and dexamethasone (DEX), across DSS‐induced acute, chronic and relapsing colitis models. Further, uhNAs reshaped the colonic immune landscape, as evidenced by the suppression of NF‐κB signaling in M1 macrophages, a shift toward regulatory M2‐like phenotype, reduced CD8^+^ T cells and neutrophils, and elevated Tregs. Overall, our study proposed a drug‐free strategy and underscored the promise to treat UC.

## 2 Results

### 2.1 Lipid Conjugation Induces Hyaluronic Acid‐Based Prodrug Self‐Assembly

Our prior efforts to conjugate therapeutics with fatty acids drove the supramolecular self‐assembly and optimized in vivo delivery of small molecular agents [24]. Here, we engineered a series of HA‐based self‐assembling chemical inducers via lipid conjugation for prolonged retention in inflamed colons. Specifically, PBA moieties were first incorporated on HA polymer to yield HA‐PBA, which was then coupled with lipidated catechols (i.e., dopamine modified monounsaturated and polyunsaturated acids, Figure 1A) at a boronate/catechol molar ratio of 1:1, yielding fatty acid‐HA conjugates (FA‐HA). ^1^H nuclear magnetic resonance and high‐resolution mass spectrometry were constructed to confirm the structures of resultant conjugates (Figures S1–S8). HA‐PBA used herein had an approximately 50% degree of PBA substitution on its carboxyl groups (Figure S2), a level selected to balance aqueous solubility and PBA density for subsequent conjugation. Energy‐dispersive X‐ray spectroscopy (EDS) mapping demonstrated the homogeneous boron (B) distribution across nanostructures (Figure 1C,D), validating the successful formation of HA‐PBA. We next assessed the affinity of HA‐PBA to lipidated catechols, OA‐DA, by exploiting a competitive binding assay in which OA‐DA competed with Alizarin Red S (ARS) for reversible phenylboronate ester bond formation with HA‐PBA. ARS was employed as a fluorescent reporter to probe boronic acid–diol interactions [25]. Free ARS did not exhibit any fluorescence, while a strong signal was observed in the ARS/HA‐PBA complex (Figure 1E). The fluorescence intensity was quenched upon the addition of OA‐DA in a dose‐dependent manner, indicating the competitive binding of OA‐DA to HA‐PBA that displaced ARS from ARS/HA‐PBA complex. Simultaneously, a corresponding change in solution color from yellow to red was also observed (Figure 1F). Moreover, there was a hypsochromic shift of λ_max_ upon addition of OA‐DA, suggesting the reorganization in chromophore packing. We then determined the binding affinity between OA‐DA and HA‐PBA. As shown in Figure S9, the association constant (K_a_) of OA‐DA/HA‐PBA complex under typical ionic conditions was 0.713 µM^−1^. Together, these results demonstrated that OA‐DA was able to conjugate with HA‐PBA via reversible boronate ester bonds. The structures of resulting compounds were also confirmed by Fourier transform infrared (FT‐IR) spectroscopy. In the FT‐IR spectrum, HA‐PBA showed enhanced absorbance at 1373 cm^−1^ (C─O/B─O stretch [26]) and a strengthened hydroxyl bond stretching (3317 cm^−1^), indicating the successful conjugation of PBA to HA polymer. The attenuation in hydroxyl bond stretching at 3317 cm^−1^ and the appearance of C─O stretching at 1283 cm^−1^ in HA‐OA, compared with that of HA‐PBA, further demonstrated the formation of boronate ester bonds [27] (Figure S10). As expected, HA‐PBA exhibited substantially greater ROS‐scavenging activity, rapidly quenching 2,2’‐Azobis(2‐amidinopropane) dihydrochloride (AAPH)‐derived peroxyl radicals within 30 min, compared to HA (*p* = 0.0014, Figure 1G), and protected Caco‐2 cells from H_2_O_2_‐induced cytotoxicity (Figure 1H), suggesting the crucial role of PBA moiety in oxidation resistance. In addition, both HA and HA‐PBA did not show any cytotoxicity.

Next, we examined the self‐assembly behaviors of FA‐HA conjugates. Compared with HA‐PBA, all of the conjugates formed nanostructures with narrow size distributions, as measured by dynamic light scattering (DLS, Figure S11A). The critical aggregation concentration (CAC) of HA‐PBA was 13.0 µg mL^−1^, whereas the CAC values were 2.6 µg mL^−1^ for uhNAs‐OA, 9.3 µg mL^−1^ for uhNAs‐LA, and 9.5 µg mL^−1^ for uhNAs‐LNA, indicating that lipid‐conjugation potentially altered intermolecular interactions and packing behavior, facilitating nanoassembly formation at low concentrations, particularly uhNAs‐OA (Figure S12). Consistent with these observations, the FA‐HA conjugates assembled into cluster‐like nanostructures with rough surfaces, as revealed by transmission electron microscopy (TEM) observation (Figure 1I). Given that OA, LA and LNA possessed the same C18 chain length, the observed differences were more likely attributable to their distinct degrees of unsaturation. Compared to OA (C18:1), the additional cis double bonds in LA (C18:2) and LNA (C18:3) were expected to impair acyl‐chain packing, thereby compromising nanoassembly stability. All of the uhNAs were negatively charged (Figure S11B), enabling dual‐specific localization to inflamed mucosa via electrostatic effects [10, 28] and HA‐CD44 interactions. Surprisingly, these nanoclusters remained sufficiently stable in the simulated gastric fluid (SGF) and simulated intestinal fluid (SIF) within 4 h (Figure 1J,K). We further investigated the pH‐ or ROS‐triggered release kinetics of uhNAs‐OA. Cy3‐DA was synthesized and incorporated into uhNAs‐OA as a model payload for assessing ROS‐responsive release (Synthesis and characterization details were provided in Figure S13). Notably, exposure to acidic buffer or H_2_O_2_ elicited a sustained release, culminating in approximately 50% cargo discharge by 72 h (Figure 1L,M). In contrast, minimal OA‐DA release was observed under physiological conditions (PBS, pH 7.4), whereas Cy3‐DA showed a modest baseline release. TEM (Figure S14) and DLS analyses (Figure S15) further confirmed the structural destabilization under acidic and oxidative conditions. Collectively, these results indicated that the boronate–catechol ester linkages were susceptible to acidic and oxidative stimuli, thereby triggering self‐immolative cleavage and spontaneous release of therapeutic agents.

### 2.2 Localization in Inflamed Colon and Enhanced M1 Macrophage Uptake

**FIGURE 2:**
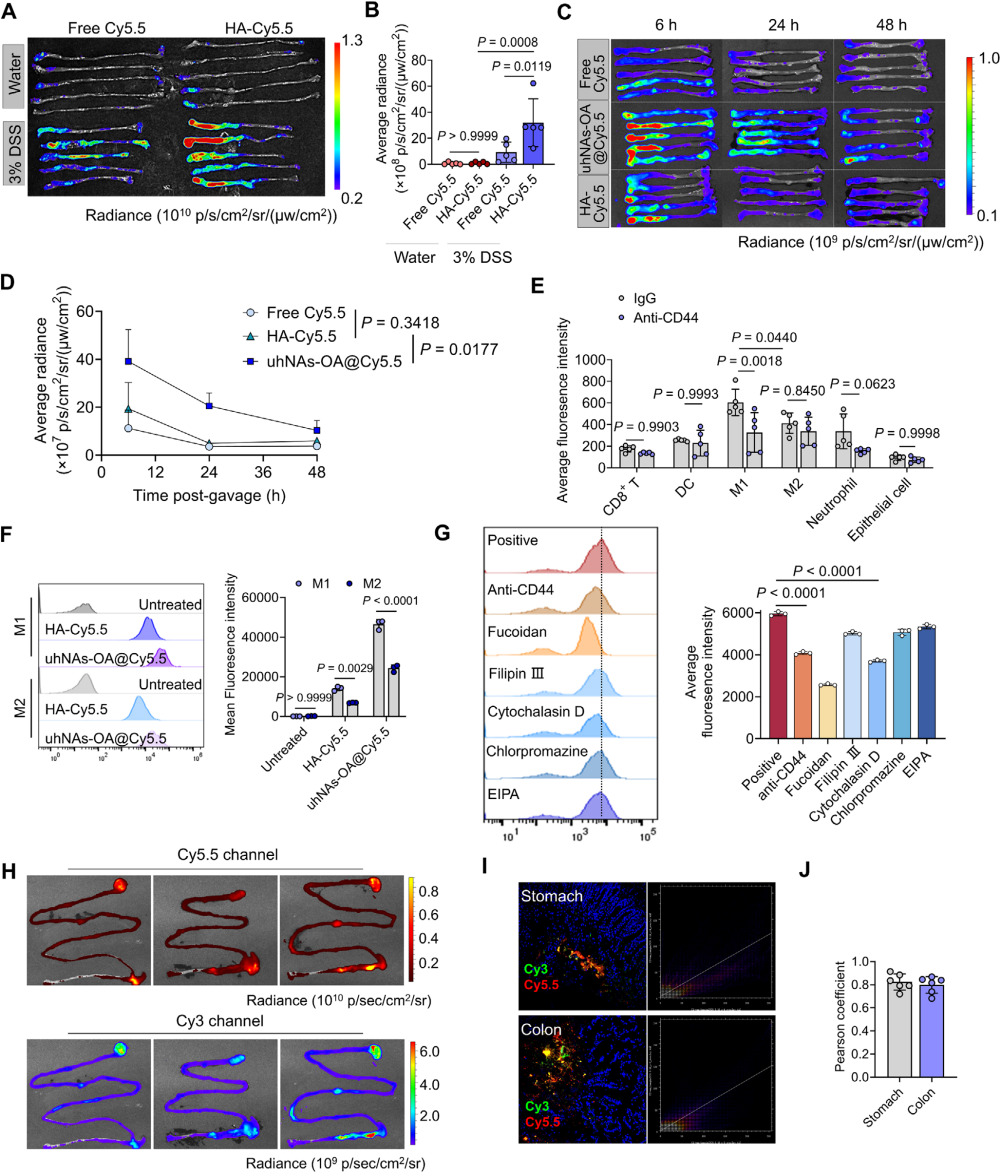
HA‐based nanoassemblies exhibited prolonged retention in the inflamed colon and preferential internalization by M1 macrophages. (A, B) C57BL/6 mice drinking 3% DSS (6 days) were treated with a single oral gavage of free Cy5.5 and HA‐Cy5.5 (0.75 mg Cy5.5 kg^−1^, n = 5). Healthy mice were included as a control. The colons and major organs were harvested and imaged using IVIS 6 h later. IVIS fluorescence images (A) and corresponding quantification (B) of colons from free Cy5.5 and HA‐Cy5.5 treated mice after 6 h of oral administration. (C, D) DSS‐treated mice received a single oral gavage of free Cy5.5, HA‐Cy5.5 and uhNAs‐OA@Cy5.5. Colons were collected at 6, 24, and 48 h post‐administration and imaged with IVIS system. Representative fluorescence images (C) and corresponding quantification of Cy5.5 signals (D) in excised colons at the indicated time points. (E) Fluorescent uhNAs‐OA@Cy5.5 quantification in CD8^+^ T cells, DCs, M1‐ and M2‐like macrophages, neutrophils and epithelial cells in the colons of colitis mice with or without anti‐CD44 antibody pretreatment, analyzed by flow cytometry. (F) Cellular uptake of HA‐Cy5.5 and uhNAs‐OA@Cy5.5 in M1‐ or M2‐like macrophages analyzed by flow cytometry. G) Cellular uptake of uhNAs‐OA@Cy5.5 in M1‐like macrophages following pretreatment with anti‐CD44 antibody or the indicated endocytic pathway inhibitors, analyzed by flow cytometry. Cells were pretreated for 30 min with anti‐CD44 antibody (1.5 µg mL^−1^), fucoidan (scavenger receptor‐mediated endocytosis inhibitor, 100 µg mL^−1^), chlorpromazine (clathrin‐mediated endocytosis inhibitor, 20 ng mL^−1^), cytochalasin D (phagocytosis inhibitor, 0.2 µM), filipin III (caveolae‐mediated endocytosis inhibitor, 500 ng mL^−1^), and EIPA (macropinocytosis inhibitor, 0.04 µM), followed by incubation with uhNAs‐OA@Cy5.5. (H) Fluorescent images of the entire GI tract at 6 h following oral administration. Cy5.5 channel: HA‐PBA‐Cy5.5; Cy3 channel: Cy3‐DA. (I) Representative confocal images showing the colocalization of HA‐PBA‐Cy5.5 and Cy3‐DA in stomach and colon tissues. (J) Pearson's correlation coefficients of Cy5.5 and Cy3 signals, quantified from two confocal images per tissue (n = 3 mice). Data were presented as the mean ± SD (n = 5 biologically independent animals for B‐E and n = 3 biologically independent samples for F, G and J). Statistical significance was examined by one‐way ANOVA with Tukey's multiple comparisons test (B and G) or two‐way ANOVA with Sidak's multiple comparisons test (D, E and F).

To determine the accumulation of HA‐based formulations in colitis tissue, mice given 3% DSS in drinking water were treated with a single oral gavage of free Cy5.5 and Cy5.5‐conjugated HA (termed as HA‐Cy5.5) and then imaged via in vivo imaging systems (IVIS). Healthy mice were included as a control. Intriguingly, both formulations showed significantly stronger fluorescence signals in inflamed colon than in normal colon at 6 h post‐administration, which might be ascribed to the disrupted colonic epithelium in DSS‐induced colitis mice (Figure 2A,B). Loss of intestinal barrier function allowed the luminal substrates to penetrate the colonic mucosa. Notably, HA‐Cy5.5 exhibited ∼3.4‐fold higher accumulation in the colitis tissue than free Cy5.5 control, while a negligible fluorescence signal was observed in major organs, including the heart, lung, liver, kidney and spleen (Figure S16), suggesting that hyaluronic acid modification enabled the accumulation of HA‐Cy5.5 in the inflamed colon. We next investigated whether nanoassembly could prolong the residence of HA‐based formulations in the inflamed colon. DSS‐induced colitic mice were orally administered free Cy5.5, HA‐Cy5.5, and uhNAs‐OA@Cy5.5, and the colons were collected at 6, 24, and 48 h post‐gavage for ex vivo imaging. As shown in Figure 2C,D, fluorescence signals gradually decreased over time in all three groups. However, uhNAs‐OA@Cy5.5 maintained higher colonic fluorescence than free Cy5.5 and HA‐Cy5.5, with appreciable signals remaining detectable at 24 and 48 h post‐administration. These results supported a more sustained retention of the nanoassembled formulations in the inflamed colon. Given that nanoformulation may reshape the cellular uptake profile of HA, we then examined the in vivo cellular distribution of HA‐based nanoformulations uhNAs‐OA@Cy5.5 in the colonic lamina propria of DSS‐induced colitic mice. As depicted in Figure 2E, M1 macrophages exhibited the highest fluorescence signals among the analysed cell populations, indicative of the preferential uptake of uhNAs‐OA@Cy5.5 by pro‐inflammatory macrophages in the inflamed colon. Consistently, in vivo blockade of CD44‐HA interaction substantially decreased the uhNAs‐OA@Cy5.5 uptake by M1 macrophages, while exerting minimal effects on M2 macrophages, dendritic cells (DCs), CD8^+^ T cells and epithelial cells (Figure 2E). A similar downward trend was also observed in neutrophils. Taken together, these results indicated that nanoassembly prolonged the retention of HA‐based formulations in the inflamed colon, whereas HA‐CD44 interactions mediated their preferential uptake by M1 macrophages.

A flow cytometry analysis showed that cells exposed to uhNAs‐OA@Cy5.5 exhibited higher signals than those treated with HA‐Cy5.5, revealing the accelerated cellular internalization following nanoassembly (Figure 2F). Pretreatment of M1 macrophages with anti‐CD44 antibodies significantly attenuated uhNAs‐OA@Cy5.5 signals, supporting the involvement of a CD44‐dependent internalization in M1 macrophages (Figure 2G). Moreover, fucoidan and cytochalasin D also reduced uhNAs‐OA@Cy5.5 uptake, suggesting that scavenger receptor‐associated endocytosis and phagocytosis additionally contributed to cellular internalization (Figure 2G), which may underlie the enhanced uptake of uhNAs‐OA@Cy5.5 relative to HA‐Cy5.5.

To further assess the in vivo stability of uhNAs following oral administration, we employed a dual‐labeling strategy by incorporating Cy3‐conjugated dopamine into uhNAs‐OA@Cy5.5 via boronate‐catechol ester bonds. At 6 h post‐gavage, the whole gastrointestinal (GI) tracts of mice were excised for IVIS fluorescence imaging. Co‐localized Cy5.5 and Cy3 signals were observed along the GI tract, indicating that uhNAs‐OA remained intact in both gastric and intestinal fluids (Figure 2H). Confocal imaging of stomach and colon sections further confirmed substantial signal overlap, with Pearson's correlation coefficients of ∼0.8 (Figure 2I,J). These data demonstrated that uhNAs maintained high structural integrity in vivo and efficiently accumulated in inflamed colon tissue.

### 2.3 uhNAs Ameliorate DSS‐Induced Colitis

**FIGURE 3:**
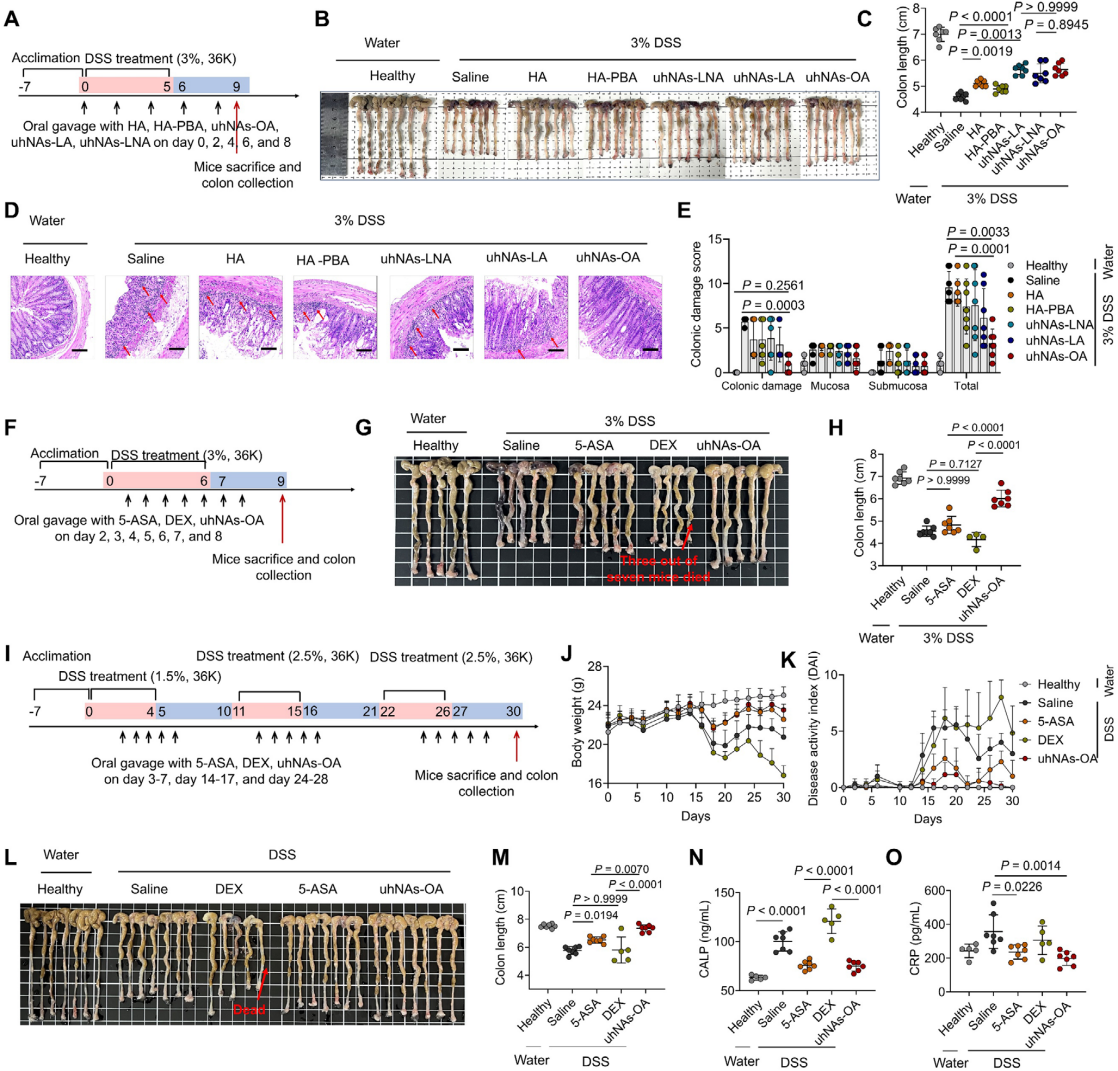
Orally administered uhNAs‐OA exerted strong efficacy in DSS‐induced acute colitis. (A) Treatment scheme for mice with acute colitis. C57BL/6 mice given 3% DSS were administered every 2 days by oral gavage of saline or 50 mg kg^−1^ (HA equivalent dose) of HA, HA‐PBA and uhNAs (‐OA, ‐LA, or ‐LNA). Healthy mice were included as a control. On day 9, mice were sacrificed and colons were harvested. Photographs of colons (B) and corresponding colon lengths (C) following various treatments. D) Representative images of H&E staining of colon tissues in each group and (E) the colon tissue sections were analyzed for colonic damage scores. Scale bar, 100 µm. (F) Treatment scheme for mice with acute colitis. C57BL/6 mice given 3% DSS were administered by daily oral gavage of saline or 30 mg kg^−1^ of 5‐ASA, 1 mg kg^−1^ DEX or 30 mg kg^−1^ uhNAs‐OA. On day 9, mice were sacrificed, colons and spleens were harvested. Healthy mice were included as a control. Colon images (G) and corresponding colon length (H) in each group. (I) Treatment schedule for mice with chronic colitis. C57BL/6 mice given 1.5–2.5% DSS were orally gavaged daily with 30 mg kg^−1^ of uhNAs‐OA, 1 mg kg^−1^ of DEX, 30 mg kg^−1^ of 5‐ASA for 5 days per cycle, over three treatment cycles. On day 30, mice were sacrificed, colon tissues and blood were collected for analysis. Body weight (J) and DAI (K) in each treatment group. Photographs of colons (L) and the corresponding colon lengths (C) following indicated treatments. Levels of colonic CALP (N) and serum CRP (O) determined by ELISA on day 30. The protein concentration of each sample was normalized to 1.5 mg mL^−1^. Data were presented as the mean ± SD (n = 5 biologically independent animals for healthy control in N‐O, and n = 7 biologically independent animals for all other panels). Statistical significance was examined by one‐way ANOVA with Tukey's (C) or Bonferroni's multiple comparisons test (H, M, N and O) or two‐way ANOVA with Tukey's multiple comparisons test (E).

Encouraged by the long‐term retention in colitis colon and M1‐targeted delivery, we next evaluated the therapeutic efficacy of uhNAs in an acute colitis model (Figure 3A). Compared with saline control, uhNAs treatment significantly protected mice against colitis, manifesting as decreased disease activity index (DAI, Figure S17), lower diarrhea scores (Figure S18), and recovery of colon length (Figure 3B,C). Importantly, uhNAs‐OA outperformed the other uhNAs, with the lowest DAI value (1.57 ± 0.98 versus 2.43 ± 0.98 in uhNAs‐LA, 3.86 ± 3.13 in uhNAs‐LNA) at the conclusion of DSS exposure. Quantitative histopathology scoring further confirmed the excellent inhibitory effect of uhNAs‐OA on colonic inflammation, evidenced by minimum crypt damage, diminished inflammatory cell infiltration and fewest colon ulcers (Figure 3D,E). Moreover, immunostaining revealed the marked suppression of NF‐κB signaling in M1‐like macrophages (Figure S19), relative to saline control, indicating the attenuated inflammatory responses. Notably, in vivo blockade of CD44‐HA interaction partially blunted the therapeutic efficacy of uhNAs‐OA, as evidenced by shortened colon length and reversal of IL‐6 reduction, with a similar trend in TNF‐α, relative to uhNAs‐OA treatment alone (Figure S20). Together with the diminished cellular uptake observed upon CD44 blockade, these findings supported a functional role for CD44‐mediated targeting in the in vivo anti‐inflammatory activity of uhNAs‐OA.

Subsequently, we evaluated the efficacy of uhNAs‐OA against two clinically established IBD drugs, 5‐ASA (30 mg kg^−1^) and DEX (1 mg kg^−1^), in DSS‐induced acute and chronic colitis models (Figure 3F,I). In mouse model of acute colitis, uhNAs‐OA exhibited more potent anti‐inflammatory activity than either 5‐ASA or DEX, maintaining colon length, normalizing spleen indices, and preventing macroscopic colonic hemorrhage (Figure 3G,H, and Figure S21), presumably due to the prolonged retention in inflamed colonic tissue and preferential uptake by M1 macrophages. Histopathological analysis revealed a remarkable alleviation in colonic damage in mice treated with uhNAs‐OA, whereas 5‐ASA and DEX showed limited improvement compared to saline control (Figure S22). Dihydroethidium staining further revealed markedly elevated ROS‐associated fluorescence signals in the colonic tissues of DSS‐treated mice, which was substantially attenuated by uhNAs‐OA treatment (Figure S23). Notably, DEX treatment induced substantial systemic toxicity, resulting in pronounced organ atrophy (i.e., 45.2% reduction in spleen weight) and mortality, while no signs of systemic side effects were observed in mice treated with uhNAs‐OA. Consistently, in mice with chronic colitis, uhNAs‐OA treatment demonstrated significant amelioration in weight loss, reduction in DAI score, and recovery of colon length, further highlighting the robust therapeutic efficacy (Figure 3J–M). Moreover, uhNAs‐OA reduced the levels of calprotectin (CALP) and C‐reactive protein (CRP, Figure 3N,O). Collectively, uhNAs‐OA exhibited promising therapeutic efficacy in both acute and chronic colitis models in a safe manner.

### 2.4 uhNAs Dampen M1 Macrophage‐Mediated Inflammation via NF‐kB Signaling Inhibition and ROS Scavenging

**FIGURE 4:**
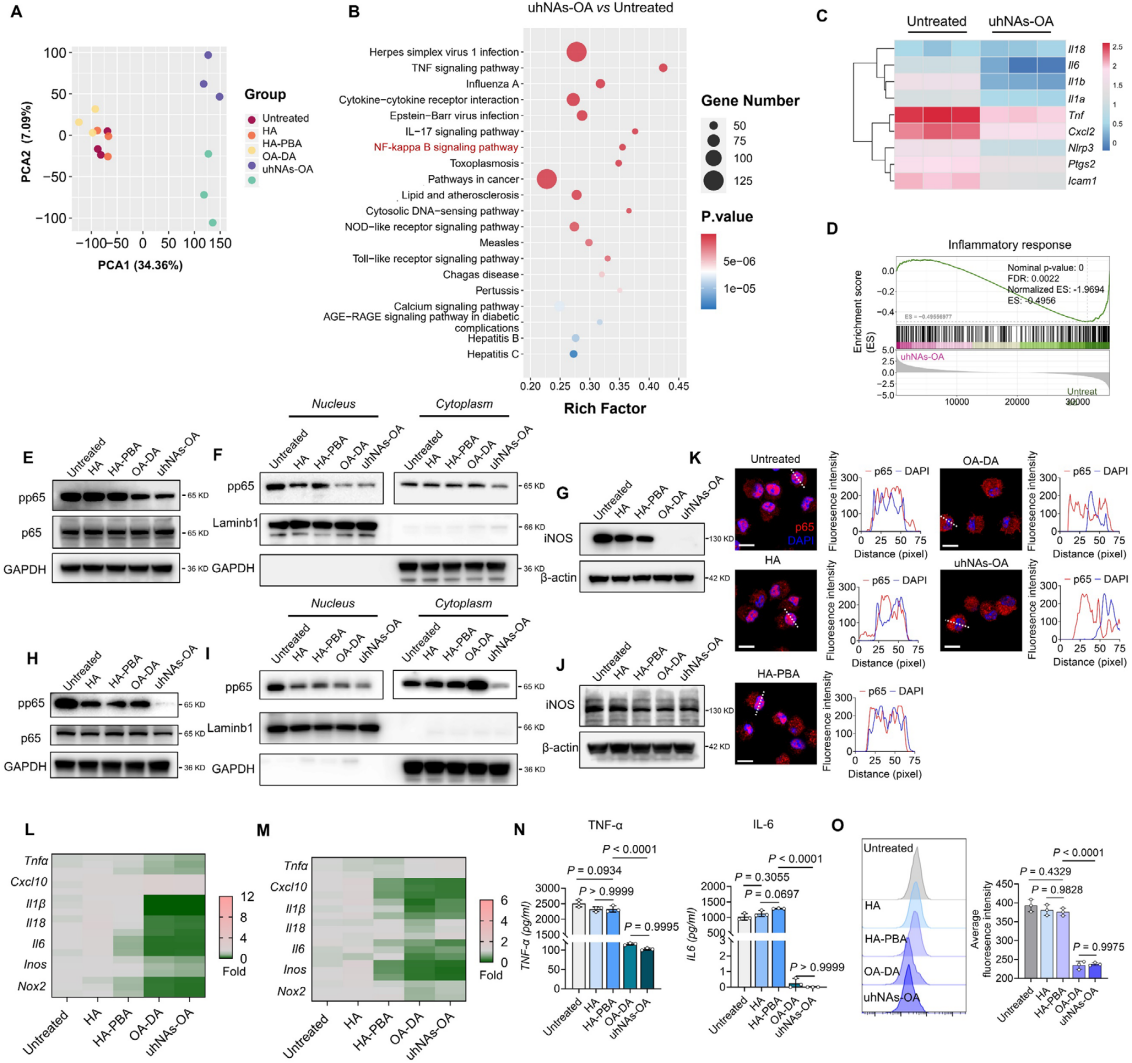
uhNAs‐OA inhibited NF‐κB activation and diminished the inflammation responses in M1‐like macrophages. (A–D) RNA‐seq analysis of IFN‐γ/LPS‐primed untreated control and drug‐treated mouse BMDMs. A) PCA was conducted on untreated control, HA, HA‐PBA, OA‐DA and uhNAs‐OA‐treated BMDMs (n = 3, each group). B) KEGG analysis of DEGs in uhNAs‐OA versus untreated control, highlighting NF‐κB signaling‐related pathways. C) Heatmap representation of NF‐κB target genes expression in untreated control and uhNAs‐OA treated BMDMs. D) Gene set enrichment analysis (GSEA) showing the reduced inflammation responses in BMDMs following uhNAs‐OA treatment. Western blot analysis of NF‐κB‐p65 phosphorylation (pp65), pp65 nuclear translocation, and iNOS expression in Raw264.7 cells (E–G) and BMDMs (H–J). Raw264.7 cells were treated with uhNAs‐OA (0.05 mg mL^−1^) for 1 h and then stimulated with IFN‐γ (50 ng mL^−1^) and LPS (100 ng mL^−1^) for 30 min. For BMDMs, cells were pre‐incubated with disulfiram for 40 min before uhNAs‐OA treatment and IFN‐γ/LPS stimulation. (K) Confocal laser scanning microscopy images identified the impaired nuclear translocation of phospho‐p65 following uhNAs‐OA exposure and IFN‐γ/LPS stimulation. NF‐κB‐p65: antibody against NF‐κB‐p65 (red), nuclei: DAPI (blue). The mRNA levels of inflammatory genes (*Tnfα*, *Cxcl10*, *Il1b*, *Il18* and *Il6*), *Inos* and *Nox2* in Raw264.7 cells (L) and BMDMs (M) following various treatments were determined by qPCR analysis. (N) Release of the indicated cytokines TNF‐α and IL‐6 was measured by ELISA. (O) Intracellular ROS generation in various treatment groups was detected with H_2_DCFDA using flow cytometry. Data were presented as the mean ± SD of three biological independent samples (n = 3). Statistical significance was examined by one‐way ANOVA with Tukey's multiple comparisons test (N and O).

To further explore the mechanism by which uhNAs alleviate intestinal inflammation, RNA‐sequencing (RNA‐seq) analyses were performed on lipopolysaccharide (LPS)/interferon‐gamma (IFN‐γ) primed bone marrow‐derived macrophages (BMDMs) following various treatments. The principal component analysis (PCA) identified a distinct cohort dispersion in the uhNAs‐OA‐treated group versus untreated control, HA‐PBA or OA‐DA‐treated group, demonstrating the global transcriptomic remodeling induced by uhNAs‐OA treatment (Figure 4A). Kyoto Encyclopedia of Genes and Genomes (KEGG) analysis of significantly differentially expressed genes (DEGs, |log_2_FC| ≥ 1, *Q* < 0.05) showed that downregulated genes were mainly involved in NF‐κB and NF‐κB‐related signaling pathways (e.g., tumor necrosis factor (TNF) and Toll‐like receptor signaling, Figure 4B). Downregulation of canonical NF‐κB effectors, including *Il6*, *Il1β*, *Tnf*, *Cxcl2*, *Nlrp3*, *Ptgs2* and *Icam1*, further corroborated the pathway inhibition (Figure 4C). The enrichments of “inflammatory response” and “positive regulation of cytokine production involved in inflammatory response” were observed in untreated control, indicating that uhNAs‐OA may inactivate NF‐κB signaling in M1 macrophages, thereby restraining inflammatory responses (Figure 4D and Figure S24). Analysis of upregulated genes in uhNAs‐OA group relative to OA‐DA alone identified the ECM‐receptor interaction pathway, associated with M1 to M2 phenotypic shift, further suggesting the enhanced efficacy of uhNAs‐OA arises from synergistic HA‐PBA and OA contributions (Figure S25).

We next quantified NF‐κB signaling‐related marker proteins in Raw264.7 and BMDMs by western blot. uhNAs‐OA substantially impeded NF‐κB‐p65 phosphorylation in response to LPS/IFN‐γ, but did not induce any changes in NF‐κB‐p65 expression (Figure 4E,H). Moreover, nuclear translocation of phospho‐p65 (Ser536) was also attenuated in both cells following uhNAs‐OA treatment (Figure 4F,I). In M1 macrophages, inducible nitric oxide synthase (iNOS) strongly increases with LPS/IFN‐γ stimulation, which is a hallmark of macrophage activation [29]. Notably, a significant reduction in iNOS expression was observed in uhNAs‐OA‐treated group (Figure 4G,J), revealing the reprogramming of M1 macrophages. Consistent with the western blot data, immunofluorescent staining confirmed that the LPS/IFN‐γ‐induced nuclear translocation of phospho‐p65 was remarkably hindered by uhNAs‐OA treatment (Figure 4K and Figure S26). Taken together, these findings indicated that uhNAs‐OA suppressed M1 macrophage‐mediated inflammatory responses through inhibition of NF‐κB signaling, as evidenced by reduced p65 phosphorylation and nuclear translocation. Indeed, quantitative PCR (qPCR) studies also detected the downregulation of *Inos* and pro‐inflammatory *Tnf*, *Cxcl10*, *Il1b*, *Il18* and *Il6* in Raw264.7 and BMDMs (Figure 4L,M), corroborating the anti‐inflammatory efficacy exerted by uhNAs‐OA. The reduction in TNF‐α and IL‐6 secretion following uhNAs‐OA treatment was further confirmed by enzyme‐linked immunosorbent assay (ELISA, Figure 4N). In addition, we observed that NADPH oxidase 2 (NOX2), an enzyme that generates ROS, was inhibited by uhNAs‐OA. As NOX2‐derived ROS is able to stimulate macrophages to produce pro‐inflammatory cytokines and chemokines [30], the reduction in Nox2 expression may lead to impaired inflammatory responses in M1 macrophages. Subsequently, using 2’,7’‐dichlorodihydrofluorescein diacetate (H_2_DCFDA, an ROS generation indicator), we measured the cellular ROS generation in response to LPS/IFN‐γ following treatments. As depicted in Figure 4O, the level of ROS was decreased in uhNAs‐OA‐treated macrophages compared with untreated cells, supporting the contribution of ROS scavenging to the anti‐inflammatory effects of uhNAs‐OA. Regrettably, HA‐PBA conjugates resulted in slight reduction in intracellular ROS, which might be ascribed to their highly polar, hydrophilic architecture that limits the endocytic uptake and subsequent intracellular access in macrophages. Of note, the downregulated genes in HA‐PBA‐treated macrophages, relative to HA‐treated control, exhibited pronounced enrichment in the Chemical carcinogenesis‐ROS pathway, suggesting the phenylboronic acid‐mediated ROS scavenging (Figure S27).

### 2.5 Covalent Engineering Potentiates the Efficacy of uhNAs‐OA Against Colitis

**FIGURE 5:**
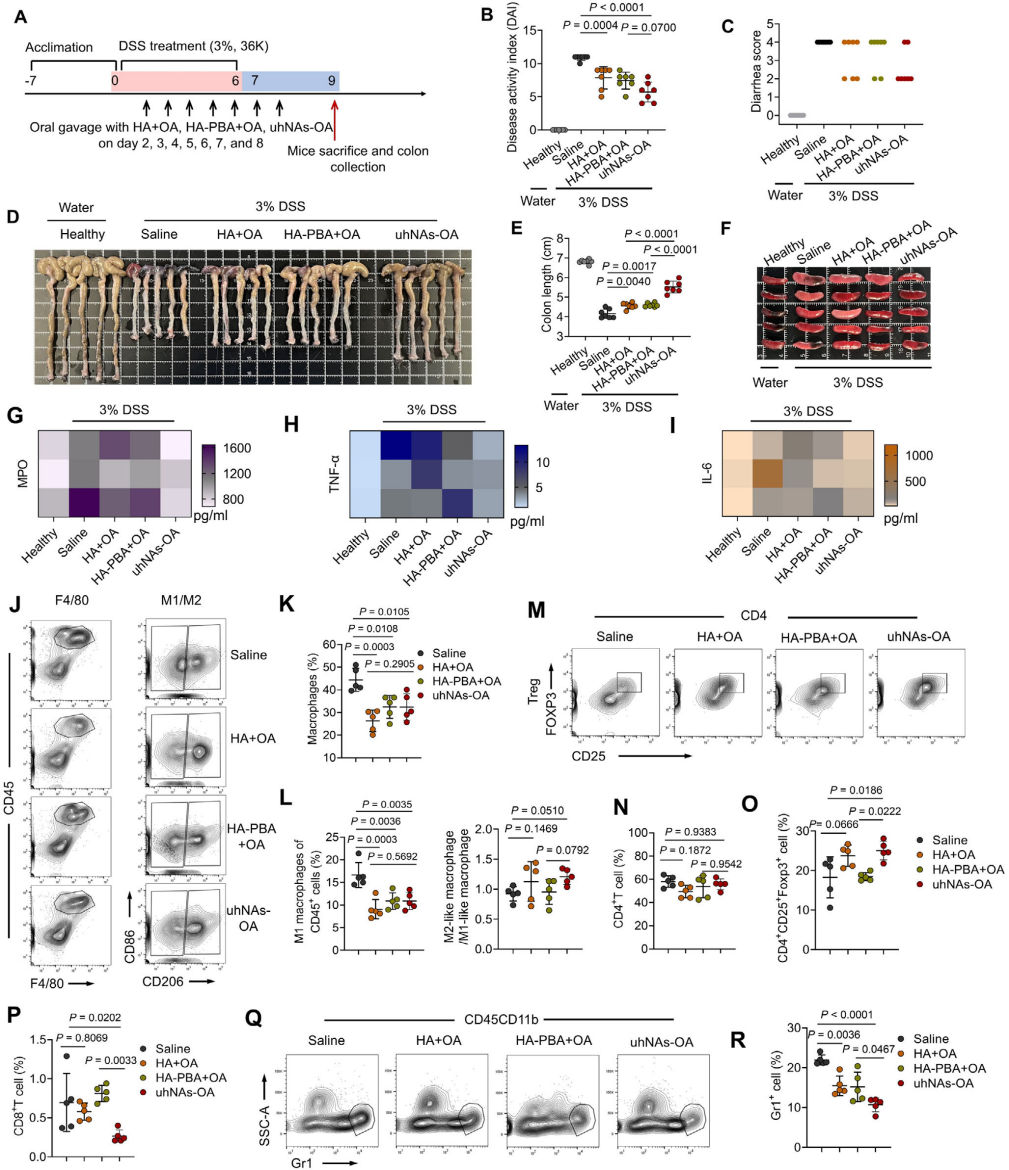
Covalent engineering of uhNAs‐OA enhanced the therapeutic efficacy in DSS‐induced acute colitis and reprogrammed the aberrant colonic immune landscape. (A) Scheme of treatment schedule. C57BL/6 mice given 3% DSS were administered by daily oral gavage of saline or 30 mg kg^−1^ of uhNAs‐OA, HA + OA or HA‐PBA + OA (equivalent dose of HA and OA of uhNAs‐OA). On day 9, mice were sacrificed, colons and spleens were harvested. DAI (B) and diarrhea scores (C) for each group on day 9. Colon images (D) and corresponding colon length (E) in each treatment group. (F) Photographs of spleens from mice with different treatments. Levels of MPO (G), TNF‐α (H) and IL‐6 (I) in colonic tissue determined by ELISA on day 9. The protein concentration of each sample was normalized to 1.5 mg mL^−1^. (J–R) Immune modulation. Colons were prepared into single cell suspension and stained with indicated antibodies for flow cytometry analysis. Representative flow cytometric plots and quantitative analysis of CD86^+^ M1‐, CD206^+^ M2‐like macrophages (J‐L), CD4^+^ T cells (N), Tregs (CD4^+^CD25^+^FOXP3^+^, M and O), CD8^+^ T cells (P) and neutrophils (CD45^+^CD11b^+^Gr1^+^, Q and R). Data were presented as the mean ± SD (n = 7 biologically independent animals for B, C and E; n = 3 biologically independent animals for G‐I; n = 5 biologically independent animals for K‐R) and statistical significance was examined by one‐way ANOVA with Tukey's multiple comparisons test.

We next compared the efficacy of uhNAs‐OA against a simple mixture of HA moieties (HA or HA‐PBA) and fatty acids OA in DSS‐induced colitis model to examine whether the covalent engineering strategy confers benefits to therapeutic outcomes (Figure 5A). Consistently, oral administration of uhNAs‐OA decreased DAI values and diarrhea scores (*p* <0.0001, Figure 5B,C), and remarkably reversed DSS‐induced colon length shortening in comparison with saline control (5.5 cm versus 4.2 cm, *p* <0.0001, Figure 5D,E). Furthermore, uhNAs‐OA alleviated colonic hemorrhage at the study endpoint (Figure 5D). In contrast, a physical mixture of HA+OA or HA‐PBA+OA treatment showed minimal therapeutic outcomes, failing to mitigate colon shortening. Also, uhNAs‐OA showed a tendency to alleviate splenomegaly (Figure 5F and Figure S28). Myeloperoxidase (MPO), a neutrophil granule enzyme, is indicative of neutrophil infiltration [31]. In the DSS‐induced colitis model, uhNAs‐OA treatment markedly reduced the level of MPO in the inflamed colon (Figure 5G), indicating the potent suppression of neutrophil recruitment and associated inflammatory damage. In parallel, uhNAs‐OA decreased the TNF‐α and IL‐6 levels in colonic tissue more significantly than other therapies (Figure 5H,I). These data suggested that covalent binding might improve the retention of therapeutics and further extend the duration of action.

Given the excellent capacity of uhNAs‐OA to target M1 macrophages and quell pro‐inflammatory cytokines production, we then mapped a reprogramming colonic immune landscape to elucidate the mechanisms of uhNAs‐OA against IBD. As depicted in Figure 5J–L, uhNAs‐OA treatment elicited a significant decrease in the frequency of macrophages compared with saline control (CD45^+^F4/80^+^CD11b^+^, 32.4% versus 44.4%, *p* = 0.0105, Figure 5K and Figure S29), and impeded M1 polarization of macrophages, shifting the M1/M2 balance toward a regulatory phenotype (Figure 5L and Figure S29). As a result, these transformations further promoted Tregs development, culminating in an enrichment of FoxP3^+^ Tregs from 18.3% (saline control) to 25.1% within the colonic lamina propria (*p* = 0.0186; Figure 5M,O, and Figure S30). Notably, total CD4^+^ T cell compartments were conserved in all treated groups versus saline control (*p* > 0.05, Figure 5N), indicating that oral administration of uhNAs‐OA triggered a shift in CD4^+^ T cell function but not in proportion. We also observed a decrease of colonic CD8^+^ cytotoxic T cells in uhNAs‐OA‐treated mice, while other treatment groups exhibited comparable CD8^+^ T cell level to saline control (Figure 5P and Figures S31 and S32). Neutrophils are the central effectors of intestinal innate immunity and involved in IBD development [32]; thus, we quantified the infiltration of neutrophils in colitis tissue. Consistent with the therapeutic benefit in acute colitis, uhNAs‐OA dramatically diminished neutrophil infiltration from 22.0% (saline control) to 10.7% (*p* < 0.0001, Figure 5Q,R, and Figure S33), thereby dampening the neutrophil‐driven inflammations and rebalancing the colon homeostasis. Overall, oral administration of uhNAs‐OA alleviated colitis by targeting activated M1 macrophages and further inhibiting inflammatory cascades, reducing CD8^+^ T cells and neutrophils while upregulating Tregs.

### 2.6 Immune Remodeling in Acute Colitis Defined by Single‐Cell RNA Sequencing

**FIGURE 6:**
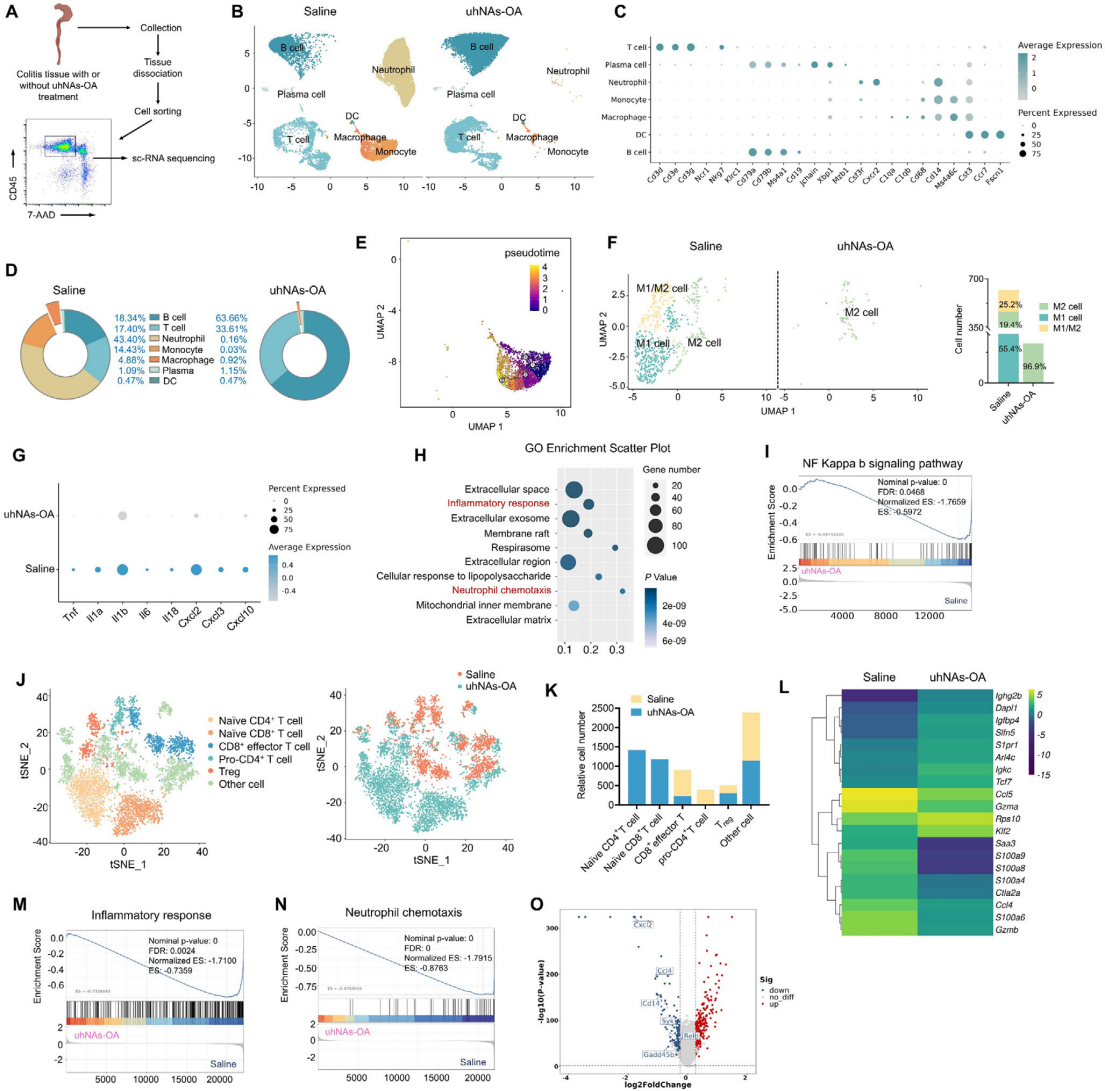
uhNAs‐OA remodeled the colonic immune landscape and downregulated inflammatory pathway signatures in DSS‐induced colitis. (A) Schematic of scRNA‐seq and sorting strategy. Mice were treated as indicated in Figure 5A. On day 9, mice were sacrificed, colons were harvested for live CD45^+^ leukocytes sorting and subsequent scRNA‐sequencing. n = 4 biologically independent samples. (B) UMAP clustering of sorted CD45^+^ leukocytes from DSS‐induced colitis mice treated with or without uhNAs‐OA. (C) Dot plot showing the canonical marker genes for each subset in (B). Color indicates the average expression of marker genes, and dot size indicates the percentage of cells expressing these genes. (D) Donut chart showing the distribution of each cell type within CD45^+^ leukocytes in two treatment conditions. (E) Trajectory analysis of monocytes and macrophages from DSS‐induced colitis mice without treatment. (F) UMAP visualization of macrophages and corresponding quantification of each identified phenotype. (G) The expression of pro‐inflammatory genes (i.e., *Tnf*, *Il1a*, *Il1b*, *Il6*, *Il18*, *Cxcl2*, *Cxcl3* and *Cxcl10*) in macrophages from saline control or uhNAs‐OA‐treated mice. (H) GO enrichment analysis of downregulated DEGs in uhNAs‐OA versus saline control specific for macrophages. (I) GSEA analysis showing the inactivation of NF‐κB signaling pathway in uhNAs‐OA‐treated group. (J) T‐cell subsets distribution in two treatment conditions was shown in t‐SNE chart: naive CD4^+^/CD8^+^ T cells, CD8^+^ effector T cells, pro‐inflammatory CD4^+^ T cells, Tregs and other cells. (K) Stacked bar plots of T‐cell subset composition across two groups. (L) Heatmap of top 20 DEGs in T cells between uhNAs‐OA and saline control. GSEA analysis showing the attenuated (M) inflammatory response and (N) Neutrophil chemotaxis in uhNAs‐OA‐treated group. (O) Volcano plot highlighting the downregulation of NF‐κB pathway‐related genes in B cells.

To comprehensively delineate the immune landscape following uhNAs‐OA treatment, we performed single‐cell RNA sequencing (scRNA‐seq) on CD45^+^ cells from colons of four DSS‐induced colitis mice with or without therapy (Figure 6A and Figure S34). Post‐quality control, 13 105 cells in uhNAs‐OA‐treated group and 14 458 cells in the saline control were collected for cluster analysis. We then identified 18 cell clusters and 7 cell populations: T cells, B cells, neutrophils, monocytes, macrophages, plasma cells and DCs, based on the uniform manifold approximation and projection (UMAP) analysis and canonical cell marker genes [33] (Figure 6B–D and Figure S35). In the saline control, neutrophils, monocytes and macrophages dominated the colonic immune landscape (62.7% of total immune cells), whereas uhNAs‐OA treatment elicited a pronounced contraction of these pro‐inflammatory populations. Intriguingly, macrophages showed gene‐expression profiles closely aligned with that of monocytes (Figure S36). Pseudotime analysis further confirmed the differentiation from infiltrating monocytes to macrophages (Figure 6E). Next, macrophage clusters were defined as M1‐like (Nos2^hi^, Il1a^hi^ and Il1b^hi^), M2‐like (Mrc1^hi^, C1qa^hi^, C1qb^hi^ and C1qc^hi^) macrophages and a continuum state of M1/M2‐like (Nos2^hi^, Arg1^hi^ and C1qb^hi^) macrophages [13, 34] (Figure 6F and Figure S37). As expected, pro‐inflammatory M1‐like phenotype dramatically decreased in uhNAs‐OA‐treated group, while anti‐inflammatory M2‐like phenotype remained stable after treatment. Consistently, macrophages from uhNAs‐OA‐treated group exhibited reduced expressions of pro‐inflammatory *Tnf*, *Il1a*, *Il1b*, *Il6*, *Il18*, *Cxcl2*, *Cxcl3* and *Cxcl10* (Figure 6G), compared with the saline control, suggesting the attenuated macrophage inflammatory activation. In addition, differentially downregulated genes in uhNAs‐OA‐treated macrophages showed enrichment for gene‐ontology (GO) terms related to “inflammatory response” and “neutrophil chemotaxis” (Figure 6H and Figure S38). Further analysis revealed the significant downregulation of “NF Kappa b signaling pathway” in uhNAs‐OA‐treated macrophages (Figure 6I). Altogether, these findings suggested that uhNAs‐OA alleviated inflammation responses by suppressing NF‐κB signaling in macrophages and curbing neutrophil recruitment.

Indeed, uhNAs‐OA treatment resulted in a remarkable expansion of T cells. To comprehensively investigate T cell composition, we performed *t*‐distributed stochastic neighbor embedding (*t*‐SNE) analysis and used cell‐type specific genes to identify T cells (Figure 6J and Figure S39). Of note, uhNAs‐OA treatment triggered large shifts in the frequence of T‐cell subsets, significantly increasing naïve T cells and regulatory Tregs while decreasing the pro‐inflammatory T‐cell phenotypes, including CD8^+^ effector T cells and Ifng^+^CD4^+^ T cells (Figure 6K). Moreover, uhNAs‐OA reshaped the T‐cell transcriptional profiles, with substantially reduced expression of *Gzma*, *Gzmb*, *Ccl4*, and *Ccl5*, and a pronounced elevation in the expression of *Klf2*, *S1pr1*, and *Tcf7*, which are involved in immune regulation and anti‐inflammatory responses in the top 20 DEGs (Figure 6L). Further profiling confirmed that inflammatory pathways were markedly downregulated in uhNAs‐OA‐treated T cells (Figure 6M and Figure S40). In addition, uhNAs‐OA curtailed T cell‐dependent neutrophil recruitment (Figure 6N and Figure S40). We then profiled B cells following uhNAs‐OA therapy and observed a substantial increase in B cells compared with saline control. Surprisingly, enrichment analysis showed attenuation of inflammatory signaling pathways, particularly the NF‐κB signaling (Figure S41), in uhNAs‐OA‐treated B cells, with *Cxcl2*, *Ccl4*, *Relb*, *Gadd45b*, *Cd14*, and *Syk* significantly downregulated (Figure 6O).

### 2.7 Sustained Colonic Immune Remodeling by uhNAs‐OA Confers Protection Against Recurrent DSS Colitis

**FIGURE 7:**
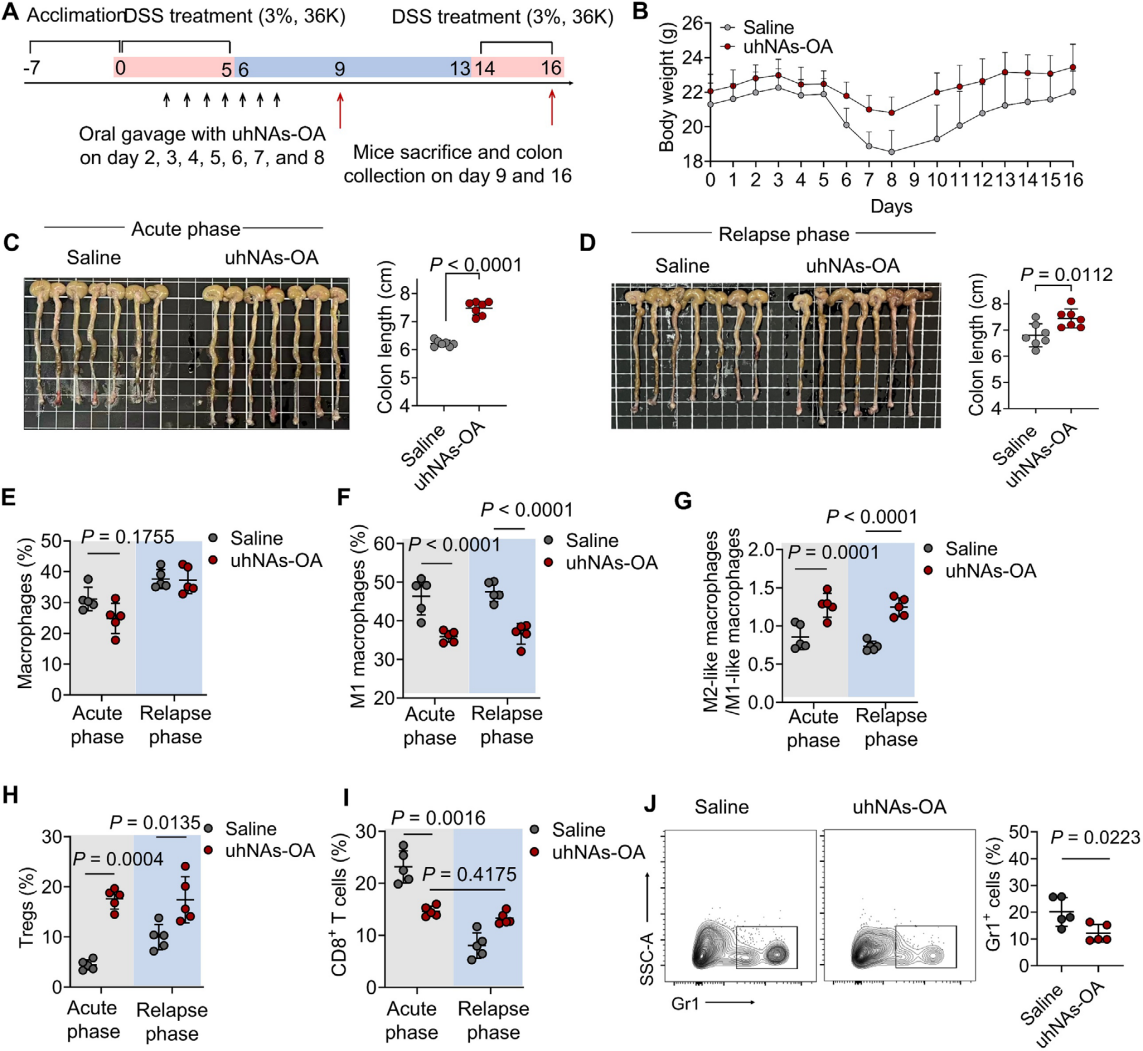
uhNAs‐OA alleviated relapsing DSS colitis and sustained immune remodeling across acute and relapse phases. (A) Treatment schedule. C57BL/6 mice were given 3% DSS for 6 days, followed by 8 days of normal drinking water, and then re‐exposed to 3% DSS for another 3 days. uhNAs‐OA (30 mg kg^−1^) was administrated daily by oral gavage on days 2–8. Mice were sacrificed on days 9 and 16, and colons were harvested. (B) Body weight in each treatment group. Digital photos and quantified lengths of colons from treated mice in the acute (C) and relapse (D) phases. (E–J) Immune modulation across acute and relapse phases. Frequencies of CD86^+^ M1‐, CD206^+^ M2‐like macrophages (E‐G), Tregs (CD4^+^CD25^+^FOXP3^+^, H), CD8^+^ T cells (I) and neutrophils (CD45^+^CD11b^+^Gr1^+^, J) in colonic tissue measured by flow cytometry. Data were presented as the mean ± SD (n = 7 biologically independent mice for C and D; n = 5 biologically independent animals for E‐J) and statistical significance was examined by Student's *t* test (C, D and J) or two‐way ANOVA with Sidak's multiple comparisons test (E‐I).

To determine whether the colonic immune remodeling induced by uhNAs‐OA was maintained upon DSS rechallenge, we established a relapse model of DSS‐induced colitis and profiled the colonic immune landscape during the acute phase and after DSS re‐exposure (Figure 7A). uhNAs‐OA attenuated disease severity at both stages, with less body weight loss and reduced colon shortening than in saline control (Figure 7B–D). Immune profiling further showed that uhNAs‐OA significantly decreased the proportion of M1 macrophages and preserved a higher M2‐like/M1‐like macrophage ratio during recurrent colitis (Figure 7E–G and Figure S42), while also inducing an increased infiltration of colonic Tregs (Figure 7H and Figure S43). Notably, uhNAs‐OA elicited a significant decrease in CD8^+^ T cells in the acute phase, and this low proportion was maintained upon DSS rechallenge (Figure 7I and Figure S44). Consistent with the results in acute colitis, uhNAs‐OA therapy markedly reduced colonic neutrophil from 20.1% (saline control) to 12.2% (*p* = 0.0223, Figure 7J). These findings strongly demonstrated the role of uhNAs‐OA in sustaining immune remodeling, protecting mice against recurrent DSS‐induced colitis.

### 2.8 Preliminary In Vivo Safety Evaluation

**FIGURE 8:**
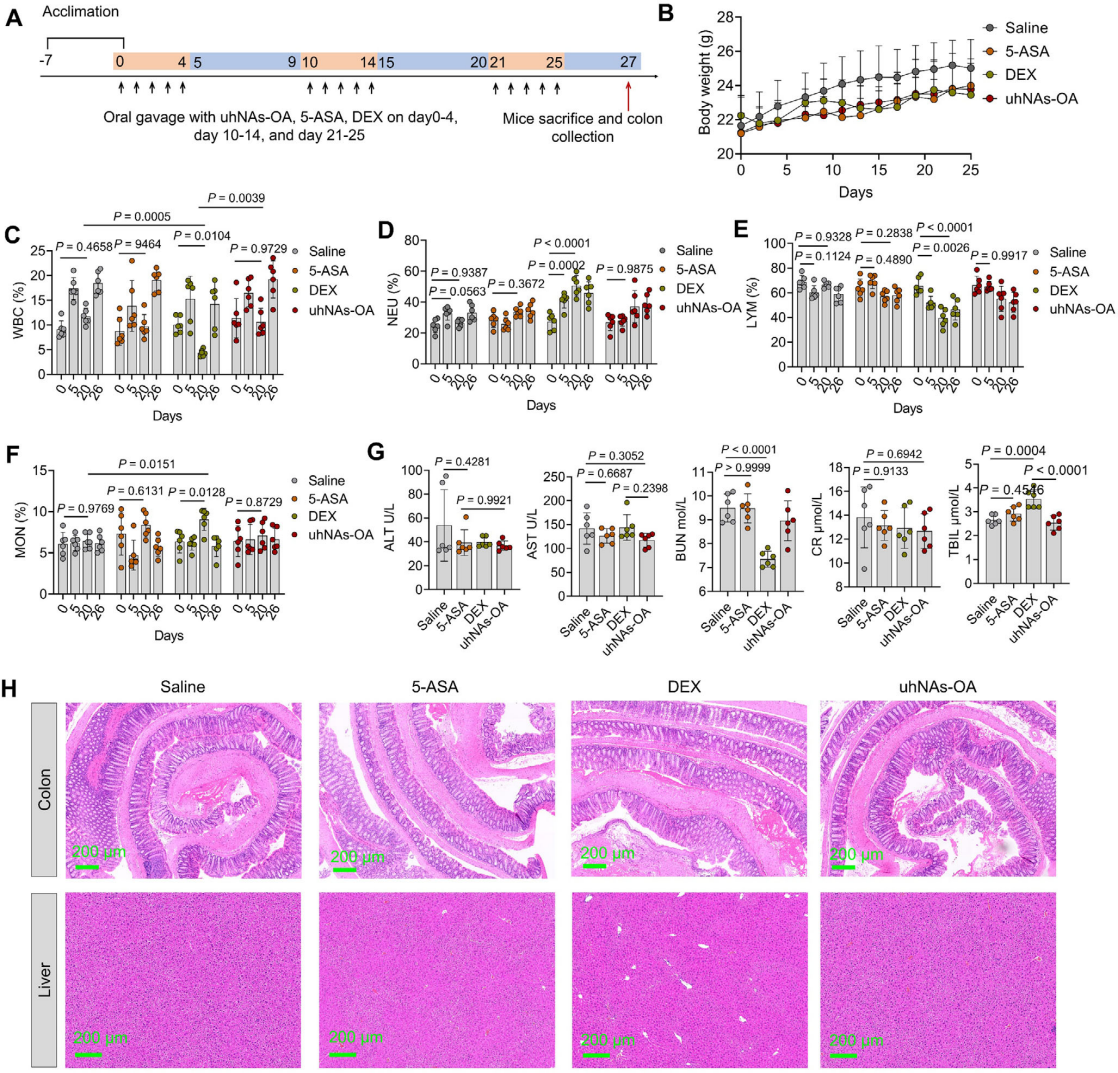
Repeated dosing of uhNAs‐OA demonstrated favourable in vivo safety in healthy mice. (A) Schematic illustration of the treatment protocol. C57BL/6 mice were orally gavaged daily with 30 mg kg^−1^ of uhNAs‐OA, 1 mg kg^−1^ of DEX, 30 mg kg^−1^ of 5‐ASA for 5 days per cycle, over three treatment cycles. On day 27, mice were sacrificed, major organs (colon, heart, liver, spleen, lung, and kidney) and blood were collected for further analysis. (B) Body weight changes in each treatment group. Hematological measurements of (C) white blood cells (WBC), (D) neutrophils (NEU), (E) lymphocytes (LYM) and (F) monocytes (MON). (G) Serum biochemical analysis of aspartate aminotransferase (AST), alanine aminotransferase (ALT), creatinine (CR), blood urea nitrogen (BUN) and total bilirubin (TBIL). (H) Haematoxylin and eosin (H&E) staining images of the colon and liver. Scale bar, 200 µm. Data were presented as mean ± SD (n = 6 biologically independent animals). Statistical significance was examined by one‐way ANOVA with Tukey's multiple comparisons test (G) or two‐way ANOVA with Tukey's multiple comparisons test (C‐F).

We then evaluated the potential toxicity of uhNAs‐OA following repeated oral administration (Figure 8A). Long‐term assessment in normal C57BL/6 mice demonstrated the biosafety profile of uhNAs‐OA, with no significant differences to saline control in body weight (Figure 8B). Hematological measurements (Figure 8C–F), serum biochemical analysis (Figure 8G) and histological evaluation (Figure 8H and Figure S45) further confirmed the systemic biocompatibility of uhNAs‐OA. By contrast, DEX treatment resulted in a significant increase in neutrophils and monocytes, along with a decrease in total white blood cells after two treatment cycles. Moreover, an increase in total bilirubin was also observed in the DEX‐treated group. These findings were consistent with the known systemic side effects of glucocorticoids. Taken together, these results indicated a minimal risk for uhNAs‐OA oral administration.

## 3 Discussion and Conclusion

Converging evidence suggests the crucial role of the monocytes‐macrophages axis in IBD pathology [35, 36, 37, 38]. Single‐cell analyses of IBD colon tissues have identified that the inflammatory macrophages originate from circulating monocytes [39]. These macrophages preferentially produce neutrophil‐recruiting chemokines (i.e., ELR^+^CXC chemokines) and pro‐inflammatory cytokines, including TNF‐α, IL‐6, IL‐1β and IL 23, thereby activating lymphocytes and stromal cells, and amplifying intestinal inflammation [2]. To date, biologic immunosuppressants such as anti‐TNF‐α [35, 39], anti‐IL‐23 [40, 41] and anti‐α4β7 [42] antibodies have demonstrated efficacy in colitis alleviation; however, therapeutic responses remain limited to a small subset of patients. Moreover, long‐term administration causes severe off‐target side effects. Given the central role of macrophages in colitis, selective reprogramming of their inflammatory state offers a tractable strategy for durable UC remission. We here describe a drug‐free therapeutic strategy that re‐educates pro‐inflammatory M1 macrophages via ROS neutralization and NF‐κB signaling attenuation.

HA has been widely used in hydrogel scaffolds [43, 44]. Here, covalent ligation of fatty acids to HA polymers via dynamic boronate‐catechol ester linkages afforded fatty acid‐HA conjugates, which could self‐assemble into well‐defined supramolecular nanostructures with narrow size distributions (Figure 1I). Importantly, OA, LA, and LNA used in this study share the same carbon‐chain length (C18) and differ primarily in the degree of unsaturation. Thus, the superior performance of uhNAs‐OA is more likely attributable to differences in acyl‐chain unsaturation and hydrophobic packing than to chain length. Consistently, uhNAs‐OA exhibited the lowest CAC among the three formulations, indicative of a stronger propensity for self‐assembly and improved stability upon dilution. We further showed that HA functionalization promoted the accumulation of HA‐based formulations in inflamed colons, whereas nanoassembly confered sustained colonic retention and preferential uptake by M1‐like macrophages. CD44 blockade significantly attenuated uhNAs‐OA internalization and partially blunted its therapeutic efficacy in acute colitis, supporting a functional contribution of CD44‐mediated targeting to the anti‐inflammatory activity of uhNAs‐OA. Notably, uhNAs‐OA remained largely intact during gastrointestinal transit, which may contribute to intestinal retention and accumulation in inflamed colon tissue. Mechanistic analyses indicated that, in M1 macrophages, uhNAs‐OA substantially scavenged excess ROS and curtailed NF‐κB signaling by downregulating p65 phosphorylation and impeding its nucleus translocation. NF‐κB and allied pathways, including TNF and Toll‐like receptor signalings, are strongly engaged in colitis pathogenesis [6, 45]; thus, such attenuation plausibly underpins the observed remission. Oral administration of uhNAs‐OA reshaped the colonic immune landscape, eliciting significant decreases in M1‐like macrophages and neutrophils with concomitant increases in Tregs. Notably, uhNAs‐OA significantly reduced CD8^+^ T cells in the acute phase, and this low proportion was maintained upon DSS rechallenge, indicating a more stable colonic immune state during recurrent inflammation. Also, uhNAs‐OA treatment reduced the levels of colonic pro‐inflammatory cytokines (TNF‐α and IL‐6), CALP and MPO, as well as serum CRP levels. A high‐resolution scRNA‐seq of colitic tissues further corroborated these shifts and mapped the myeloid, B and T‐cell programs, highlighting the attenuation of NF‐κB signaling in macrophages and B cells, and a *Klf2/S1pr1*‐linked quiescent/regulatory reprogramming in T cells (Figure S46). Upregulation of *Klf2/S1pr1* in Foxp3^+^Ccr7^+^Sell^+^ (cTreg‐like) and Tcf7^+^Ccr7^+^Sell^+^ (naïve T) T cell populations suggested the enhanced lymphocyte egress and recirculation [46, 47] (Figure S39 and S46), consistent with improved mucosal immune homeostasis following uhNAs‐OA treatment. Together, these coordinated effects conferred the robust efficacy of uhNAs‐OA against DSS‐induced actue colitis. Compared with physical mixtures of HA (or HA‐PBA) and OA, uhNAs‐OA markedly restored colon length, decreased DAI values and alleviated colonic damage, underscoring covalent engineering as the key determinant of therapeutic efficacy. In addition, uhNAs‐OA exerted more potent therapeutic efficacy than other clinical IBD drugs, 5‐ASA and DEX in both acute and chronic colitis, which might be ascribed to the selective accumulation at colitis sites. The efficacy observed in chronic and relapsing DSS colitis further supported its capacity to sustain colonic immune remodeling during recurrent intestinal inflammation. Although DSS‐induced colitis is initiated by epithelial injury, macrophage‐targeted suppression of ROS and NF‐κB signaling observed here may also be relevant to adaptive immune‐driven colitis, warranting direct evaluation in complementary models, such as CD4^+^CD45RB^high^ T‐cell‐transfer colitis. Repeated oral administration in healthy mice further supported the favourable preliminary safety profile of uhNAs‐OA. Following oral administration, uhNAs‐OA was expected to disassemble and undergo enzymatic or metabolic degradation, whereas components not retained at inflamed colonic sites were likely to be eliminated through the gastrointestinal tract. Nevertheless, its long‐term biodistribution, degradation and clearance warrant further investigation.

Of note, PBA constitutes the core boronic acid moiety of 4‐borono‐L‐phenylalanine (Steboronine), a compound approved in Japan for clinical boron neutron capture therapy (BNCT) of unresectable or recurrent head‐and‐neck cancers; in parallel, both HA and OA are well recognized for their biocompatibility, collectively supporting the translational promise of uhNAs‐OA. In addition, uhNAs were generated via self‐assembly, and independent batches prepared under identical HA‐PBA/lipidated catechol feed ratios, solvent compositions and mixing conditions exhibited comparable particle sizes and polydispersity indices, supporting batch‐to‐batch reproducibility at the laboratory scale. Although the present study was strengthened by the inclusion of chronic and relapse colitis models, together with repeated‐dose safety evaluation, further studies are still required to assess long‐term efficacy and safety in more clinically relevant settings and to support scalable manufacturing. Nevertheless, our study demonstrated an oral, drug‐free nanotherapeutics that accumulated in the inflamed colon and M1 macrophages, rebalanced the colonic immune landscape, thereby exerting robust anti‐inflammatory responses against UC. Given the potent attenuation in NF‐κB signaling, our strategy described here may provide a facile and promising therapy for other NF‐κB‐driven inflammatory diseases.

## 4 Experimental Section

### 4.1 Cell Lines and Cell Culture

Cancer cells Caco2 (RRID: CVCL_0025) were purchased from the Cell Bank of the Chinese Academy of Sciences (Shanghai, China) and cultured in RPMI‐1640 medium supplemented with 10% fetal bovine serum, penicillin (100 units mL^−1^) and streptomycin (100 µg mL^−1^). The murine monocyte/macrophage cell line Raw264.7 (RRID: CVCL_0493) was a gift from Professor Jianfeng Tu's group, Zhejiang Provincial People's Hospital and cultured in DMEM containing 10% heat‐inactivated fetal bovine serum, 1% penicillin and streptomycin. Both cell lines were routinely tested free of mycoplasma using a Mycoplasma Stain Assay Kit (C0296, Beyotime). BMDMs were harvested from the bone marrow of male C57BL/6 mice aged six to eight weeks according to the reported protocol and cultured in DMEM containing 10% heat‐inactivated fetal bovine serum, 1% penicillin‐streptomycin, and 50 ng/mL murine recombinant M‐CSF. BMDMs were also tested free of mycoplasma. All cells were cultured in a humid atmosphere with 5% CO_2_ at 37°C.

### 4.2 Mice

C57BL/6 mice (male) were purchased from Vital River Laboratory Animal Technology Co., Ltd. (Beijing, China). All animal studies were conducted in accordance with the National Institute Guide for the Care and Use of Laboratory Animals. The experimental protocols were approved by the Ethics Committee of Zhejiang Cancer Hospital (2025‐05‐024). The mice were housed in pathogen‐free settings at 25°C ± 2°C, 40%–60% humidity and under a normal 12‐h light/dark cycle.

Full experimental details including compound synthesis, preparation and characterization of uhNAs, ROS scavenging, cellular uptake and colonic accumulation, endocytic uptake by intestinal immune cells and the corresponding efficacy against actue colitis with or without CD44 blockade, therapeutic efficacy investigation in DSS‐induced colitis mouse models, immune profiling, in vivo stability of uhNAs, biosafety evaluation, sc RNA sequencing, and mechanistic studies are described in Supporting Information.

## Author Contributions

**Jianqin Wan**: conceptualization, methodology, data curation, investigation, validation, formal analysis, funding acquisition, project administration, writing – original draft, writing – review and editing. **Andi Ma**: investigation, validation, formal analysis, visualization, software. **Yiqiong Zhang**: investigation, validation, formal analysis. **Minghao Zhang**: validation, visualization, formal analysis. **Yifeng Lin**: methodology, investigation, formal analysis. **Xiaoling Zhang**: investigation. **Hongjun Li**: conceptualization, methodology, writing – review and editing. **Weidong Han**: conceptualization, methodology, funding acquisition, writing – review and editing, project administration.

## Conflicts of Interest

The authors declare no conflicts of interest.

## Supporting information




**Supporting File**: advs77872‐sup‐0001‐SuppMat.docx.

## Data Availability

The data that support the findings of this study are available from the corresponding author upon reasonable request.
