# Supplementary material for: Assembled Hyaluronic Acid Therapeutics for Targeting M1 Macrophages and Regulating Immune Homeostasis for Ulcerative Colitis Treatment
Source: Adv Sci (Weinh). 2026 Sep 27:e77872. Online ahead of print. doi: 10.1002/advs.77872 (PMC13616341; doi:10.1002/advs.77872)
Supplement: Supplementary file 1 [file ADVS-9999-e77872-s001.docx]

Supporting Information

Assembled hyaluronic acid therapeutics for targeting M1 macrophages and regulating immune homeostasis for ulcerative colitis treatment

Jianqin Wan, Andi Ma, Yiqiong Zhang, Minghao Zhang, Yifeng Lin, Xiaoling Zhang, Hongjun Li, Weidong Han*

J. Wan, A. Ma, Y. Zhang, M. Zhang, Y. Lin, X. Zhang, W. Han

Zhejiang Cancer Hospital

Hangzhou Institute of Medicine (HIM)

Chinese Academy of Sciences

Hangzhou, Zhejiang 310022, China

H. Li

Liangzhu Laboratory

Zhejiang University Medical Center

Hangzhou, Zhejiang 311121, China

* To whom correspondence should be addressed.

E-mail: hanwd@zjcc.org.cn

**This PDF file includes:**

Supplementary text

Figures S1 to S46

**Materials**

Oleic acid, linoleic acid and linolenic acid were purchased from J&K Chemical (Shanghai, China) and Tokyo Chemical Industry (TCI). Dopamine hydrochloride was purchased from Sigma Aldrich (USA). HA sodium salt was supported by Bloomage (Shandong Freda Biopharm Co., Ltd.). 2’,7’-dichlorodihydrofluorescein diacetate (H_2_DCFDA), Alizarin Red S (ARS) and 2,2’-Azobis(2-amidinopropane) dihydrochloride (AAPH) were obtained from MedChemExpress (USA). EIPA (S9849), Filipin III (S7402), Cytochalasin D (S8184), and Chlorpromazine (S5749) were purchased from Selleck (USA). Cyanine3 NHS ester (#41020) and Cyanine5.5 NHS (#57020) ester were purchased from Lumiprobe. Lycopersicon Esculentum (Tomato) Lectin (DyLight® 488, DL-1174-1) was purchased from Vector Laboratories (USA). Pepsin and trypsin were purchased from GLPBIO (Shanghai, China). Recombinant Mouse CSF-1/M-CSF Protein (M-CSF), mIL4 and lipopolysaccharides (LPS) were purchased from Abclonal (Wuhan, China). Anti-mouse IL-6 and TNF-α Valukine ELISA kits were purchased from R&D Systems (USA). Mouse myeloperoxidase/MPO (70-EK2133/2) and C-Reactive Protein/CRP (70-EK294/2-96) ELISA Kit was purchased from multi sciences (China). Mouse CALP (Calprotectin, E-EL-M1143) ELISA Kit was purchased from Elabscience (China). Frozen Section ROS Stain Kit (G4817) was purchased from Solarbio (China). Fixable Viability Stain 510 (#564406) was purchased from BD biosciences. All of other compounds and solvents were purchased from commercial suppliers (Sigma-Aldrich, Tokyo Chemical Industry (TCI), or J&K Chemical) and used without further purification.

**Antibodies**

The primary antibodies used for immunoblotting and immunofluorescence were against phospho-NF-κB p65 (Ser536, 93H1, Lot: 19, #3033, Cell Signaling Technology, 1000×), iNOS (D6B6S, Lot: 7, #13120, Cell Signaling Technology, 1000×), NF-κB p65 (Lot: 00163260, #10745-1-AP, Proteintech, 1000×), Lamin B1 (Lot:00170022, #12987-1-AP, Proteintech, 5000×), GAPDH (Lot: 23011098, #81640-5-RR, Proteintech, 5000×) and β-actin (Lot: F117411, #AB0035, Abways, 10000×). The secondary antibodies used for immunoblotting and immunostaining were Goat anti-Rabbit IgG, HRP linked (H+L, Lot: J805428P, #WD-GAR007, Dawen Biotech, 3000×), donkey anti-rabbit IgG, Alexa Fluor™ 555 (H+L, Lot: 3029771, #A31572, Thermo Fisher Scientific, 500×). The antibodies used for flow cytometry analysis were against CD45 (Lot: 4071400, #557659, clone: 30-F11, BD Biosciences), CD11b (Lot: B410918, #101206, clone: M1/70, Biolegend), F4/80 (Lot: B416422, #123137, clone: BM8, Biolegend), CD206 (Lot: B411647, #141712, clone: C068C2; Lot: B435518, #141708, clone: C068C2, Biolegend), CD86 (Lot: 4088509, #560582, clone: GL1, BD Biosciences), CD11c (Lot: 3352999, #563057, clone: HL3, BD Biosciences), CD3e (Lot: 3297771, #553063, BD Biosciences), CD8a (Lot: 3229734, #553032, clone: 53-6.7, BD Biosciences; Lot: B429680, #100722, clone: 53-6.7, Biolegend), CD4 (Lot: 4236104, #740007, clone: RM4-5, BD Biosciences), CD326 (Ep-CAM, Lot: B368767, #118241, clone: G8.8, Biolegend), CD25 (Lot: B420707, #102012, clone: PC61, Biolegend), FOXP3 (Lot: B409977, #126406, clone: MF-14, Biolegend), Ly-6G/Ly-6C (Gr-1, Lot: B442389, #108442, clone: RB6-8C5, Biolegend) and CD16/32 (Lot: B431984, #101302, clone: 93, Biolegend). The antibody used for CD44 blockade (anti-CD44 antibody, #CL8944LE, clone KM81) was purchased from Cedarlane.

**General methods for organic synthesis**

All reactions were performed in a dry atmosphere. Thin layer chromatography (TLC) was performed on silica gel 60 F_254_ precoated aluminum sheets (Merck) and visualized using fluorescence quenching. Chromatographic purification was performed using flash column chromatography on silica gel (neutral, Qingdao Haiyang Chemical Co., Ltd). ^1^H nuclear magnetic resonance (NMR) spectra were recorded on a Bruker 400 spectrometer at 400 MHz in CDCl_3_, DMSO-d6 and D₂O. Chemical shifts were calibrated to the residual solvent peak or tetramethylsilane (= 0 ppm). Multiplicities are abbreviated as follows: s = singlet, d = doublet, t =triplet, m = multiplet.

**Methods**

**Synthesis of HA-PBA**

Hyaluronic acid was prepared by dialyzing HA sodium salt (320 kDa) against 0.01 M HCl for 24 h and collected by a freeze-drying method. Then, hyaluronic acid (0.0003125 mmol, 100 mg) and 4-(bromomethyl) phenyl boronic acid (1.5825 mmol, 340 mg) were dissolved in 25 mL of dry dimethyl sulfoxide (DMSO), and mixed with cesium carbonate (0.7913 mmol, 257.8 mg). The mixture was stirred at 75 °C for 24 h and then dialyzed against menthol: DI water (v/v, 1:1), menthol: DI water (v/v, 1:3) and DI water. The resulting HA-PBA powder (108.1mg, 91.8%) was obtained through a lyophilization step.

HA:

^1^H NMR (400 MHz, D_2_O): δ 4.48 (s, 2H), 3.94-3.25 (m, 10H), 1.91 (s, 3H).

HA-PBA:

^1^H NMR (400 MHz, D_2_O): δ 7.80-7.73 (m, 1H), 7.47 (t, *J* = 8.0, 1H), 5.28 (d, *J* = 64.0, 1H), 4.44 (d, *J* = 36.0, 2H), 3.77-3.27 (m, 10H), 1.95-1.87 (m, 3H).

**Synthesis of HA-Cy5.5 and HA-PBA-Cy5.5**

To a solution of HA (0.0000625 mmol, 20 mg) and Cy5.5-NHS (0.0026 mmol, 2 mg) in 4.5 mL of DMSO and water (v/v, 1.5:3) were added N,Ndiisopropylethylamine (DIEA, 0.0528 mmol, 6.8 mg). The mixture was stirred 45 °C overnight and the solution was dialyzed against menthol: DI water (v/v, 1:1), menthol: DI water (v/v, 1:3) and DI water. The resulting HA-Cy5.5 powder was obtained through a lyophilization step. HA-PBA-Cy5.5 conjugate was synthesized using the same method. HA-PBA (0.000053 mmol, 20 mg) and Cy5.5-NHS (0.00222 mmol, 1.71 mg) were used as starting materials.

**Synthesis of Cy3-DA**

To a solution of Cy3-NHS (0.00779 mmol, 5.0 mg) and dopamine hydrochloride (0.00935 mmol, 1.77 mg) in 2 mL of anhydrous DMF were added N,Ndiisopropylethylamine (DIEA, 0.0187 mmol, 2.4 mg). The mixture was stirred at room temperature under nitrogen atmosphere for 5 h. And the organic layer was evaporated under vacuum. The residue was further purified by flash column chromatography on silica gel (DCM: MeOH = 7.5:1) to obtain Cy3-DA conjugate (3.9 mg, 73.6%).

^1^H NMR (400 MHz, CDCl_3_): δ 8.65 (d, *J* = 32.0, 2H), 8.30 (t, *J* = 16.0, 1H), 7.77 (t, *J* = 4.0, 1H), 7.59 (d, *J* = 8.0, 2H), 7.42-7.39 (m, 4H), 7.28-7.23 (m, 2H), 6.56 (d, *J* = 8.0, 1H), 6.51 (d, *J* = 4.0, 1H), 6.45-6.34 (m, 3H), 4.06 (t, *J* = 8.0, 2H), 3.60 (s, 3H), 3.12-3.07 (m, 2H), 2.01-1.99 (m, 2H), 1.95-1.93 (m, 2H), 1.65 (d, *J* = 4.0, 16H), 1.51-1.50 (m, 2H).

**Synthesis of OA-NHS**

To a solution of oleic acid (OA, 3.54 mmol, 1 g) and N-Hydroxysuccinimide (NHS, 3.89 mmol, 0.45 g) in 2 mL of anhydrous DCM were added 1-ethyl-3-(3-dimethylaminopropyl) carbodiimide (EDC, 4.25 mmol, 0.66 g). The mixture was stirred at 45°C for 4 h and subsequently washed with 5% citric acid, saturated NaHCO_3_ and brine. The organic layer was dried over anhydrous Na_2_SO_4_, filtered, and evaporated under vacuum. The residue was further purified by flash column chromatography on silica gel (only DCM) to obtain OA-NHS conjugate (1.2407 g, 92.4%).

^1^H NMR (400 MHz, CDCl_3_): δ 5.39-5.32 (m, 2H), 2.83 (s, 4H), 2.60 (t, *J* = 8.0, 2H), 2.02 (d, *J* = 4.0, 4H), 1.78-1.71 (m, 2H), 1.42-1.27 (m, 20H), 0.88 (t, *J* = 8.0, 3H).

**Synthesis of LA-NHS**

LA-NHS was synthesized following the same protocol with OA-NHS. Linoleic acid (LA, 3.57 mmol, 1 g) and NHS (3.93 mmol, 0.45 g) were used as starting materials. Yield: 83.9 %, 1.1302 g.

^1^H NMR (400 MHz, CDCl_3_): δ 5.40-5.28 (m, 4H), 2.82 (s, 4H), 2.76 (t, *J* = 8.0, 2H), 2.59 (t, *J* = 8.0, 2H), 2.06-2.01 (m, 4H), 1.77-1.69 (m, 2H), 1.41-1.24 (m, 14H), 0.88 (t, *J* = 8.0, 3H).

**Synthesis of LNA-NHS**

LNA-NHS was synthesized following the same protocol with OA-NHS. Linolenic acid (LNA, 3.59 mmol, 1 g) and NHS (3.95 mmol, 0.450 g) were used as starting materials. Yield: 77.0 %, 1.0372 g.

^1^H NMR (400 MHz, CDCl_3_): δ 5.42-5.26 (m, 6H), 2.82-2.74 (m, 8H), 2.59 (t, *J* = 8.0, 2H), 2.10-2.02 (m, 4H), 1.77-1.69 (m, 2H), 1.36-1.29 (m, 8H), 0.98-0.87 (m, 3H).

**Synthesis of OA-DA**

To a solution of OA-NHS (0.79 mmol, 300 mg) and dopamine hydrochloride (0.66 mmol, 124.91 mg) in 4 mL of anhydrous DMF were added N,Ndiisopropylethylamine (DIEA, 1.32 mmol, 170.25 mg). The mixture was stirred at room temperature under nitrogen atmosphere for 5 h and then washed with 5% citric acid and brine. The organic layer was dried over anhydrous Na_2_SO_4_, filtered, and evaporated under vacuum. The residue was further purified by flash column chromatography on silica gel (DCM: MeOH = 20:1) to obtain OA-DA conjugate (257.2 mg, 93.5%).

^1^H NMR (400 MHz, DMSO-d_6_): δ 8.68 (d, *J* = 40.0, 2H), 7.78 (t, *J* = 4.0, 1H), 6.62-6.40 (m, 3H), 5.32 (t, *J* = 8.0, 2H), 3.17-3.12 (m, 2H), 2.03-1.96 (m, 6H), 1.45 (t, *J* = 8.0, 2H), 1.24 (s, 22H), 0.85 (t, *J* = 8.0, 3H).

HRMS: calcd for [C_26_H_44_NO_3_]^+^ = 418.3316; obsd: 418.3309.

**Synthesis of LA-DA**

LA-DA was synthesized following the same protocol with OA-DA. LA-NHS (0.80 mmol, 300 mg) and dopamine hydrochloride (0.66 mmol, 125.3 mg) were used as starting materials. Yield: 94.7%, 260.6 mg.

^1^H NMR (400 MHz, DMSO-d_6_) : δ 8.66 (d, *J* = 40.0, 2H), 7.77 (t, *J* = 4.0, 1H), 6.62-6.40 (m, 3H), 5.38-5.27 (m, 4H), 3.18-3.12 (m, 2H), 2.73 (t, *J* = 8.0, 2H), 2.01 (t, *J* = 4.0, 6H), 1.46 (t, *J*=8.0, 2H), 1.35-1.24 (m, 16H), 0.86 (t, *J* = 8.0, 3H).

HRMS: calcd for [C_26_H_42_NO_3_]^+^ = 416.3159; obsd: 416.3154.

**Synthesis of LNA-DA**

LNA-DA was synthesized following the same protocol with OA-DA. LNA-NHS (0.80 mmol, 300 mg) and dopamine hydrochloride (0.67mmol, 125.9 mg) were used as starting materials. Yield: 95.4%, 262.6 mg.

^1^H NMR (400 MHz, DMSO-d_6_): δ 8.68 (d, *J* = 40.0, 2H), 7.78 (t, *J* = 4.0, 1H), 6.62-6.40 (m, 3H), 5.39-5.24 (m, 6H), 3.17-3.12 (m, 2H), 2.79-2.72 (m, 4H), 2.07-2.00 (m, 6H), 1.49-1.42 (m, 2H), 1.32-1.19 (m, 10H), 0.94-0.84 (m, 3H).

HRMS: calcd for [C_26_H_40_NO_3_]^+^ = 414.3003; obsd: 414.2997.

**FT-IR spectroscopy**

All samples were fully dried in a desiccator to prevent undesirable oxygen-containing peaks. The FT-IR spectra were recorded on a JASCO FTIR-4600 spectrometer.

**Preparation and characterization of uhNAs**

The lipidated catechols (OA-DA, LA-DA and LNA-DA) dissolved in 100 µL of DMSO were complexed with HA-PBA at a molar ratio of 1: 1 in 900 µL of DI water upon ultrasonication to prepare unsaturated fatty acid-HA nano-assemblies (uhNAs). lipidated catechols nano-assemblies were prepared using the same method. Prior to use, the solution was further dialyzed against DI water to remove DMSO. The hydrodynamic diameters (D_H_), distribution and zeta potential of resulting uhNAs and lipidated catechols nano-assemblies were determined using a Malvern Nano-ZS90 instrument (Malvern, UK) at 25°C. The morphology of uhNAs were observed with transmission electron microscopy (TEM, Talos L120C, Thermo Scientific) at an acceleration voltage of 120 kV following staining with 2 wt% aqueous uranyl acetate solution.

**Stability of uhNAs in SGF and SIF**

uhNAs (0.1 mg mL^-1^, HA equivalent) were incubated in SGF (pH = 2, PBS containing 3.2 mg mL^-1^ pepsin) or SIF (pH = 6.8, PBS containing 10 mg mL^-1^ pepsin) at room temperature. Dynamics light scattering analysis was utilized to monitor the particle size and polydisperse index (PDI) during incubation.

**Fluorescence Competitive Binding Assay**

ARS was mixed with HA-PBA (7.6 × 10^-4^ mmol) at a molar ration of 1: 1 in PBS solution (pH 7.4), and then serial molar ratios of OA-DA (0.1-2.5 equiv. of HA-PBA) were added into the mixture. Variation in the fluorescence signal at 596 nm was recorded by fluorescence spectrophotometer (Agilent, USA). Soluble ARS and mixture of HA-PBA/OA-DA served as the control.

**Evaluation of ROS scavenging**

HA or HA-PBA (1 mg mL^-1^, HA equivalent) in DI water was pre-incubated with a solution of a peroxy radical-generating reagent AAPH (1 mM) for 5 or 30 min at 37 °C. The solution was then incubated with ICG (4 µg mL^-1^), and the absorption of ICG at 779 nm was recorded by UV-vis spectrometry. AAPH solution without HA or HA-PBA was included as a control.

***In vitro* drug release**

To evaluate the pH-responsive release kinetics, 2 mL of uhNAs-OA (0.1 mg mL^-1^, HA equivalent) was dialyzed against 10 mL of PBS buffers (pH 7.4 or 6.2) containing 50% DMSO at 37 °C. At pre-determined times, samples were collected and analyzed with reversed-phase high performance liquid chromatography (RP-HPLC).

To evaluate ROS-triggered release kinetics, Cy3-conjugated dopamine was incorporated into uhNAs-OA *via* boronate-catechol ester bonds at an OA-DA/Cy3-DA molar ratio of 2:1. Subsequently, 3 mL of Cy3-labelled uhNAs-OA (0.04 mg mL^-1^, Cy3 equivalent) was dialyzed against 12 mL of PBS buffers (pH 7.4, 10% DMSO) with or without 200 µM H_2_O_2_ at 37 °C. Samples were collected at pre-determined times and analyzed by UV-vis spectrometry.

**CCK-8 assay**

Caco2 cells (2 × 10^4^ per well) were seeded into 96-well plates and cultured at 37°C overnight. Subsequently, the cultured medium in each well was discarded and replenished with H_2_O_2_ (200µM), HA or HA-PBA (1 mg mL^-1^, HA equivalent) with or without H_2_O_2_ (200µM). Untreated cells served as the control. Following 24 h-incubation, 10 μL of CCK-8 solution was added to each well for another 2-h incubation. The absorbance was measured at 450 nm using a microplate reader (Multiskan FC, Thermo Fisher Scientific). Cell viability was calculated as follows: cell viability (%) = (absorbance of drug formulations treated well - absorbance of blank well)/ (absorbance of control well- absorbance of blank well) × 100%.

**Cellular uptake evaluation**

BMDMs were seeded into 12-well plates and stimulated with IL4 (20 ng mL^-1^) or LPS (100 ng mL^-1^) and IFN-γ (50 ng mL^-1^) for 48 h to induce an M2-like or M1-like polarization state, respectively. Subsequently, M1 or M2-like macrophages were treated with HA-Cy5.5 and uhNAs-OA@Cy5.5 (400 nM, Cy5.5 equivalent) for 4 h at 37°C, respectively. After incubation, cells were harvested, rinsed with cold PBS and analyzed by flow cytometry (LSRFortessa, BD). For another assay, Raw264.7 cells (140 × 10^4^ per well) were seeded into 12-well plates and stimulated with LPS (100 ng mL^-1^) and IFN-γ (50 ng mL^-1^) overnight to induce an M1-like polarization state. Then, M1-like macrophages were pretreated with 25 ng mL^-1^ CD44-HA inhibitor (MCE, HY-149897) or 1.5 µg mL^-1^ anti-CD44 antibody (clone KM81, Cedarlane) for 30 min at 4 °C and then incubated with uhNAs-OA@Cy5.5 (400 nM, Cy5.5 equivalent) for another 3 h at 37°C. Following treatment, cells were harvested and analyzed with flow cytometry. To investigate the potenrial endocytosis pathway involved in the cellular uptake of uhNAs-OA@Cy5.5, cells were also pretreated with specific endocytosis inhibitors for 30 min before nanoparticle incubation, for instance, chlorpromazine (20 ng mL^-1^), cytochalasin D (0.2 µM), filipin III (500 ng mL^-1^), fucodian (100 µg mL^-1^), and EIPA (0.04 µM).

**Immunofluorescence and confocal microscopy**

BMDMs and Raw264.7 cells were seeded into glass-bottom dishes and cultured at 37°C overnight. For BMDMs, cells were pretreated with disulfiram (a pyroptosis inhibitor, 20 µM) for 40 min and then replaced with different agents (including HA, HA-PBA, OA-DA and uhNAs-OA, 0.05 mg mL^-1^, HA equivalent) in fresh culture medium. Following 1-h incubation, cells were primed with LPS (100 ng mL^-1^) and IFN-γ (50 ng mL^-1^) for 30 min. Subsequently, cells were fixed with 4% paraformaldehyde for 15 min, permeated with 0.5% Triton X-100 for 15 min and blocked with 5% BSA in PBS for 1 h at room temperature. Following incubation with NF-κB p65 antibody overnight at 4 °C and Alexa Fluor™ 555 donkey anti-rabbit IgG at room temperature for 2 h, cells were stained with DAPI and observed using a confocal microscope (STELLARIS 8 STED, Leica). Disulfiram treatment was not performed on Raw264.7 cells. All other treatments and staining were performed using the same protocol.

**Immunoblotting**

BMDMs and Raw264.7 cells were seeded into 6-well plates and treated as described in the method of “Immunofluorescence and confocal microscopy”. Cells were harvested and lysed with RIPA buffer containing protease inhibitor, phenylmethanesulfonyl fluoride (PMSF, 100 ×, Beyotime), and phosphatase inhibitor cocktails on ice. The protein concentration was determined using a BCA Protein Assay Kit (Beyotime). All samples were run on an 4%-20% SDS-PAGE gel (GenScript), followed by a transfer onto polyvinylidene difluoride membrane. The membranes were then blocked with 5% BSA in tris-buffer/0.1% Tween-20 (TBST) for 1 h at room temperature, and incubated with primary antibodies, including anti phospho-NF-κB p65 rabbit mAb, anti-NF-κB p65 rabbit mAb, anti-iNOS rabbit mAb, anti-Lamin B1 rabbit mAb and anti-GAPDH rabbit mAb and anti-β-actin rabbit mAb overnight at 4 °C. After complete washes with TBST, membranes were incubated with HRP-conjugated anti-Rabbit IgG for another 2 h at room temperature. The final protein bands were visualized on the the Amersham ImageQuant 800 (Cytiva, Tokyo, Japan). Disulfiram treatment was not performed on Raw264.7 cells. All other treatments and staining were performed using the same protocol.

**RNA extraction and quantitative RT-PCR (qPCR)**

BMDMs and Raw264.7 cells were seeded into 6-well plates and cultured at 37°C overnight. For BMDMs, cells were pretreated with disulfiram (a pyroptosis inhibitor, 20 µM) for 1 h and then cultured with different drug formulations, including HA, HA-PBA, OA-DA and uhNAs-OA, 0.05 mg mL^-1^, HA equivalent for 3 h. Next, cells were primed with LPS (100 ng mL^-1^) and IFN-γ (50 ng mL^-1^) for another 4 h. After complete washes with PBS, total RNA was extracted by an RNA-quick purification kit (ESscience, RN001). All RNA samples were reverse transcribed into cDNA using HiScript IV All-in-One Ultra RT SuperMix (Vazyme, R433-01) for qPCR. Quantitative PCR (qPCR) was performed with SupRealQ Purple Universal SYBR qPCR Master Mix (U+, Vazyme, Q412-02) on a quantstudio5-2 machine (Thermo Fisher Scientific). Disulfiram treatment was not applied to Raw264.7 cells. All other treatments and staining were performed using the same protocol. The primers used for qPCR are listed as follow:

Tnfα-Fwd: 5’-CAGGCGGTGCCTATGTCTC-3’

Tnfα-Rev: 5’-CGATCACCCCGAAGTTCAGTAG-3’

Il6-Fwd: 5’-CTGCAAGAGACTTCCATCCAG-3’

Il6-Rev: 5’-AGTGGTATAGACAGGTCTGTTGG-3’

Cxcl10-Fwd: 5’-GGAGTGAAGCCACGCACAC-3’

Cxcl10-Rev: 5’-ATGGAGAGAGGCTCTCTGCTGT-3’

Il1β-Fwd: 5’-GCAACTGTTCCTGAACTCAACT-3’

Il1β-Rev: 5’-ATCTTTTGGGGTCCGTCAACT-3’

Il18-Fwd: 5’-GACTCTTGCGTCAACTTCAAGG-3’

Il18-Rev: 5’-CAGGCTGTCTTTTGTCAACGA-3’

Inos-Fwd: 5’-GTTCTCAGCCCAACAATACAAGA-3’

Inos-Rev: 5’-GTGGACGGGTCGATGTCAC-3’

Nox2-Fwd: 5’-AGTGCGTGTTGCTCGACAA-3’

Nox2--Rev: 5’-GCGGTGTGCAGTGCTATCAT-3’

**Intracellular ROS measurement**

2’,7’-Dichlorodihydrofluorescein diacetate (H_2_DCFDA, MCE) was used to detect intracellular ROS generation induced by LPS and IFN-γ. Raw264.7 cells (200 × 10^4^ per well) were seeded into 6-well plates and cultured overnight. Cells were treated with HA, HA-PBA, OA-DA and uhNAs-OA at a HA equiv. concentration of 0.05 mg mL^-1^ for 3 h. Then, the cultured medium in each well was discarded and replenished with fresh culture medium containing LPS (100 ng mL^-1^) and IFN-γ (50 ng ng mL^-1^). Following another 4-h incubation, H_2_DCFDA (10 μM) in fresh culture medium was added and further incubated for 30 min at 37 °C. After washing, cells were collected and subjected to flow cytometry.

**Bulk RNA sequencing**

To perform RNA sequencing analysis, BMDMs cells were seeded into 6-well plates and cultured at 37°C overnight. Cells were pretreated with disulfiram for 1 h and then cultured with different drug formulations (including HA, HA-PBA, OA-DA and uhNAs-OA, 0.05 mg mL^-1^, HA equivalent) for 3 h. Next, cells were primed with LPS (100 ng mL^-1^) and IFN-γ (50 ng mL^-1^) for another 4 h. Following PBS washing, Total RNA was isolated from BMDMs and purified using TRIzol reagent (Invitrogen, Carlsbad, CA, USA) following the manufacturer's procedure. The RNA amount and purity of each sample was quantified using NanoDrop ND-1000 (NanoDrop, Wilmington, DE, USA). The subsequent library construction and sequencing process was commissioned to LC-Bio Technology CO., Ltd. (Hangzhou, China). Briefly, the average insert size for the final cDNA library was 300 ± 50 bp and the 2×150 bp paired-end sequencing (PE150) was performed on an illumina Novaseq™ 6000 (LC-Bio Technology CO., Ltd., Hangzhou, China) following the vendor's recommended protocol. Then sequence quality was also verified using fastp. We used HISAT2 (https://ccb.jhu.edu/software/hisat2) to map reads to the reference genome of mus_musculus Ensembl_v112. The differentially expressed mRNAs were selected with fold change > 2 or fold change < 0.5 and with parametric F-test comparing nested linear models (p value < 0.05) by R package edgeR (https://bioconductor.org/packages/release/bioc/html/edgeR.html).

**DSS-induced colitis models and treatment**

**i) DSS-induced acute colitis**

Male C57BL/6 mice were allowed to acclimate for 1 week prior to study initiation. Acute colitis model was induced by 3% (w/v) DSS (36 to 50 kDa; MP Biomedicals) administration for 5-6 days. Control healthy mice were provided with sterile water only. For the prevention setting, mice given 3% DSS were dosed by oral gavages of HA, HA-PBA, uhNAs-OA, uhNAs-LA, or uhNAs-LNA (50 mg kg^-1^, HA equivalent) every other day (five doses total). Diseased and healthy mice treated with saline were included as controls. Fecal bleeding and consistency of mice in each group were monitored daily. On the last day, mice were sacrificed and the colon was harvested. Colon length was measured and rinsed with cold PBS. Then, two 0.5 cm segments of distal colon were collected for histological evaluation and immunofluorescence staining. For the therapeutic setting, 30 mg kg^-1^ of uhNAs-OA, 30 mg kg^-1^ of HA+OA mixture (equivalent dose of HA and OA of uhNAs-OA), HA-PBA+OA mixture (equivalent dose of HA and OA of uhNAs-OA), 1 mg kg^-1^ of DEX, 30 mg kg^-1^ of 5-ASA^1, 2, 3^ was administrated daily starting on the third day up to day 8 *via* oral gavage. Diseased and healthy mice treated with saline were included as controls. Fecal bleeding and consistency of mice were monitored. On the last day, mice were sacrificed, the colon and spleen tissues were collected for further analysis. Colon length was measured and rinsed with cold PBS. A piece of 0.5 cm colon of represented mice in each group was harvested for hematoxylin and eosin (H&E) analysis. The remaining colon tissues were used for immune cell profiling determination by flow cytometry. Furthermore, spleens were photographed and weighed.

**ii) DSS-induced relapse colitis**

Mice were given 3% (w/v) DSS (36 to 50 kDa; MP Biomedicals) for 6 days, followed by 8 days of normal drinking water, and then re-exposed to 3% DSS for another 3 days. Meanwhile, mice were daily oral gavaged with uhNAs-OA (30 mg kg^-1^) from day 2 to day 8. On day 9 and 16, mice were sacrificed, and colons were collected for further analysis, including colon length measurement and immune cell profiling determination.

**iii) DSS-induced chronic colitis**

To establish chronic colitis, mice were subjected to three cycles of DSS treatment, consisting of 1.5% (w/v) DSS (36 to 50 kDa; MP Biomedicals) for 5 days followed by 6 days of normal drinking water in the first cycle, and 2.5% (w/v) DSS for 5 days followed by 6 days of normal drinking water in the second and third cycles. Since day 3, the mice were daily oral gavaged with 30 mg kg^-1^ of uhNAs-OA, 1 mg kg^-1^ of DEX, 30 mg kg^-1^ of 5-ASA for 5 days, and treatment was given for three cycles. On the last day, mice were sacrificed, and colons were collected for further analysis, including colon length measurement and cytokines detection.

**Histological analyses and immunostaining**

Hematoxylin and eosin (H&E) staining and immunostaining of colonic tissue sections were carried out by Servicebio (Wuhan, China). Briefly, the excised 0.5 cm colon sections were fixed in 4% (v/v) paraformaldehyde and dehydrated with gradient alcohol, and then embedded in paraffin. 4 µm-thick slices of colon tissues were prepared and stained with H&E. The severity of colonic damage was scored as reported^1^: 0, normal; 1, (crypt abscess and goblet cell loss); 2, (crypt loss, 10%-50%); 3, (crypt loss, 50%-90%); 4, (complete crypt loss but intact colon epithelium); 5, (ulceration, width <10 crypts); 6, (ulceration, width ≥ 10 crypts). Inflammatory infiltrates were scored independently in the mucosa (0, none; 1, mild; 2, moderate; 3, severe), and submucosa (0, none; 1, mild; 2, moderate; 3, severe) and the final scores (epithelial damage + inflammation) ranged from 0 to 12. For immunostaining, tissue sections were stained with antibodies (F4/80, CD86 and NF-κB p65, Servicebio) and DAPI. Finally, colon sections were observed under a fluorescence microscope (3DHISTECH, Pannoramic MIDI).

**Disease activity index**

The DAI was scored as the following criteria: 1) bloody stools: 0, none; 1, light bleeding; 2, moderate bleeding; 3, heavy bleeding; 2) stool consistency: 0, hard; 2, soft; 4, diarrhea; 3) body weight loss: 0, none; 1, < 5%; 2, 5%-10%; 3, 10%-15%; 4, > 15%.

**Cytokine detection**

Raw264.7 cells (200 × 10^4^ per well) were seeded into 6-well plates and cultured at 37 °C overnight. Cells were treated as described in the method of “Intracellular ROS measurement”. Then, the medium supernatants were collected and the levels of TNF-α and IL-6 were detected using ELISA kit (R&D, USA) according to the manufacturer’s instructions.

The levels of TNF-α, IL-6 and MPO in colitis tissue were also determined using ELISA kit according to the manufacturer’s instructions. Briefly, C57BL/6 mice given 3% DSS were dosed by daily oral gavages of physical mixture of HA+OA, HA-PBA+OA (equivalent dose of HA and OA of uhNAs-OA), and uhNAs-OA at 30 mg kg^-1^ for 7 days. Diseased and healthy mice treated with saline were included as controls. On the last day, colon tissues were harvested and homogenized using a SWE-FP PLUS homogenizer (Servicebio) in NP-40 lysis buffer supplemented with PMSF at 4 °C. All tissue homogenates were incubated on ice for another 30 min. Then, tissue homogenates were centrifuged for 10 min at 12000 rpm and the supernatants were collected for cytokine detection. The protein concentration in supernatant was determined using a BCA Protein Assay Kit (Beyotime).

The levels of colonic CALP and serum CRP from DSS-induced chronic colitis model were also determined using ELISA kit, following the same protocol.

**IVIS imaging**

To determine the accumulation of HA-based formulations in colitis tissue, diseased mice (given 3% DSS for 6 days) or heathy mice were treated with a single oral gavage of free Cy5.5 and Cy5.5-conjugated HA (termed as HA-Cy5.5) at a dose of 0.75 mg Cy5.5 kg^-1^. After 6 h, mice were sacrificed and organs including colon, heart, liver, spleen, lung and kidney were excised and imaged using an IVIS imaging system with a Cy5.5 filter channel and an exposure time of 0.2 s. To assess the retention of uhNAs-OA in colitis tissue, diseased mice (given 3% DSS for 6 days) received a single oral dose of free Cy5.5, HA-Cy5.5 or uhNAs-OA@Cy5.5 at equivalent fluorescence emission intensities. At 6, 24 and 48 h after administration, mice were sacrificed, and colons were harvested and imaged using an IVIS imaging system with a Cy5.5 filter channel and an exposure time of 0.5 s.

***In vivo* stability evaluation**

To assess the *in vivo* stability of uhNAs following oral administration, a dual-labelling strategy was used, in which Cy3-conjugated dopamine was incorporated into uhNAs-OA@Cy5.5 *via* boronate-catechol ester bonds. C57BL/6 mice given 3% DSS were treated with a single oral gavage of uhNAs-OA@Cy5.5/Cy3. At 6 h post-gavage, the whole gastrointestinal (GI) tracts were excised and imaged using the Cy3 channel (Cy3-DA) and Cy5.5 channel (HA-PBA-Cy5.5), respectively. Then, frozen sections of stomach and colon tissue were prepared to investigate the colocalization efficiency of Cy3 and Cy5.5 signals by CLSM. Pearson’s correlation coefficient (PCC) was analyzed using ImageJ.

**CD44 blockade and cellular distribution of uhNAs-OA@Cy5.5 in inflamed tissue**

To assess the effect of CD44 blockade on the uptake of uhNAs-OA by immune cell subsets in inflamed colon tissue, mice were intravenously injected with anti-CD44 antibody (clone KM81, Cedarlane, 20 µg/mouse) 2 h before oral gavage with uhNAs-OA@Cy5.5. After *ex vivo* imaging, colon tissues were cut into pieces and incubated in intestinal epithelial dissociation solution supplemented with 5% fetal bovine serum, 1 mM DTT and 5 mM EDTA at 37 °C for 40 min. After complete washes with RPMI-1640, the colon pieces were minced into ~1 mm pieces and further incubated in digestion solutions containing 5% fetal bovine serum and collagenase D IV (1 mg mL^-1^) at 37°C for 30 min. Subsequently, the digestion solutions were filtered through a 70 µm cell strainer to obtain single-cell suspension. Next, cells were stained with live/dead cell staining dye, Fixable Viability Stain 510, at room temperature for 15 min, blocked with anti-mouse CD16/32 antibody at 4 °C for 10 min, and then stained with APC-Cy7 anti-mouse CD45, FITC anti-mouse CD11b, BV421 anti-mouse F4/80, APC anti-mouse CD206, PE-Cy7 anti-mouse CD86, BV605 anti-mouse CD11c, PE anti-mouse CD8a, and BV650 anti-mouse CD326 (EpCAM) at 4 °C for 20 min. After washes with PBS, cells were analyzed by flow cytometry. DCs were gated as CD45^+^CD11c^+^ cells; CD8^+^ T cells were gated as CD45^+^CD8a^+^ cells; M1 macrophages were gated as CD45^+^CD11b^+^F4/80^+^CD86^+^ cells; M2 macrophages were gated as CD45^+^CD11b^+^F4/80^+^CD206^+^ cells; epithelial cells were gated as CD45^-^CD326^+^ cells. For neutrophil, cells were stained with APC-Cy7 anti-mouse CD45, FITC anti-mouse CD11b and BV650 anti-mouse Ly-6G/Ly-6C, and were gated as CD45^+^CD11b^+^ Ly-6G/Ly-6C ^+^ cells. The cellular uptake of uhNAs-OA@Cy5.5 was quantified as the mean fluorescence intensity of Cy5.5 within each gated population.

**CD44 blockade and therapeutic efficacy of uhNAs-OA in acute colitis**

To evaluate the effect of CD44 blockade on uhNAs-OA treatment in acute colitis, mice were intravenously injected with anti-CD44 antibody (clone KM81, Cedarlane; 20 µg per mouse) every 2 days on days 2, 4, and 6, 2 h before oral gavage with uhNAs-OA@Cy5.5. Since day 2, the mice were daily oral gavaged with 30 mg kg^-1^ of uhNAs-OA for 6 days. On day 8, mice were euthanized and colon tissues were harvested for colon length measurement and cytokines (TNF-α and IL6) detection.

**Colonic ROS detectiom**

Frozen colon sections were prepared and stained with DHE (G4817, Solarbio) to detect ROS, with nuclei counterstained using DAPI. Finally, colon sections were observed under a confocal microscope (STELLARIS 8 STED, Leica).

***In Vivo* Immune Cell Staining for Flow Cytometry**

C57BL/6 mice given 3% DSS were dosed by daily oral gavages of physical mixture of HA+OA, HA-PBA+OA (equivalent dose of HA and OA of uhNAs-OA), and uhNAs-OA at 30 mg kg^-1^ for 7 days. Diseased mice treated with saline were included as controls. On the last day, colons were harvested and prepared into single-cell suspensions.

For lymphoid cells analysis, cells were stained with live/dead cell staining dye, Fixable Viability Stain 510, for 15 min, blocked with anti-mouse CD16/32 antibody for 10 min, and then stained with APC-Cy7 anti-mouse CD45, PE anti-mouse CD3e, BV421 anti-mouse CD4, PE-Cy7 anti-mouse CD8a, APC anti-mouse CD25, and AF488 anti-mouse FOXP3 for 20 min. After complete washes, cells were analyzed by flow cytometry. CD8^+^ T cells were gated as CD45^+^CD3e^+^CD8a^+^ cells; CD4^+^ T cells were gated as CD45^+^CD3e^+^CD4^+^ cells; Tregs were gated as CD45^+^CD3e^+^CD4^+^CD25^+^FOXP3^+^ cells. For myeloid cells analysis, cells were stained with live/dead cell staining dye, Fixable Viability Stain 510, for 15 min, blocked with anti-mouse CD16/32 antibody for 10 min, and then stained with APC-Cy7 anti-mouse CD45, FITC anti-mouse CD11b, BV421 anti-mouse F4/80, APC anti-mouse CD206, PE-Cy7 anti-mouse CD86, and BV650 anti-mouse Ly-6G/Ly-6C for 20 min. After complete washes, cells were analyzed by flow cytometry. M1 macrophages were gated as CD45^+^CD11b^+^F4/80^+^CD86^+^ cells; M2 macrophages were gated as CD45^+^CD11b^+^F4/80^+^CD206^+^ cells; Neutrophils were gated as CD45^+^CD11b^+^ Ly-6G/Ly-6C ^+^ cells.

**Single-cell RNA sequencing**

Colitic mice were dosed by daily oral gavages of saline or uhNAs-OA (30 mg kg^-1^). Following seven doses, colon tissues were harvested, minced and dissociated (n = 4 biological replicates/group). Flow sorting was performed to obtain live CD45^+^ cells (F2104503, multi sciences) and the single-cell suspensions were added to the 10x Chromium chip, with the expectation of capturing 10,000 cells. After library construction, an Illumina NovaSeq 6000 sequencing system with a minimum depth of 20,000 reads per cell was used for RNA sequencing. Sequencing data generated by Illumina were converted to FASTQ format using bcl2fastq software and then compared with reference genome using CellRanger software. Cells with < 500 genes detected, or with mitochondrial gene expression > 25% were excluded from further analysis. Marker genes were defined as those expressed in > 10% of cells in each cluster, with *P* ≤ 0.01 and log₂ fold change ≥ 0.26. Cell-type annotation was performed using literature-based marker genes and validated gene signatures. GO and KEGG enrichment analyses were carried out on the differentially expressed genes from each cluster (identified *via* FindAllMarkers), using the hypergeometric test to compare each cluster’s gene list against all other clusters.

**Safety evaluation**

Male C57BL/6 mice were allowed to acclimate for 1 week prior to study initiation. Subsequently, mice were randomly divided into four groups and orally gavaged daily with saline, 30 mg kg^-1^ of uhNAs-OA, 1 mg kg^-1^ of DEX, 30 mg kg^-1^ of 5-ASA for 5 days per cycle over three cycles. The body weight was recorded over 25 days. Blood samples were collected at predetermined time points for hematology assays. On day 26, mice were euthanized and major organs including heart, liver, spleen, lung, kidney, and colon were harvested for histopathological analysis. Moreover, blood samples were also obtained for serum biochemical analysis.

**Statistical analysis**

Statistical analyses were performed with GraphPad Prism 8.0, and the *P* < 0.05 were considered statistically significant. Data are presented as the means ± standard deviation (SD). Differences among multiple groups involving one variable were analyzed using one-way ANOVAs with Tukey’s or Bonferroni’s multiple comparisons test. Differences among multiple groups across more than one variable were analyzed using two-way ANOVAs with Tukey’s or Sidak’s multiple comparisons test. Student’s *t* test was used to for two-group comparisons.

**Figure S1**. ^1^H NMR spectrum of HA in D_2_O.

**Figure S2**. ^1^H NMR spectrum of HA-PBA in D_2_O.

**Figure S3**. ^1^H NMR spectrum of OA-NHS in CDCl_3_.

**Figure S4**. ^1^H NMR spectrum of LA-NHS in CDCl_3_.

**Figure S5**. ^1^H NMR spectrum of LNA-NHS in CDCl_3_.

**Figure S6**. **A**, ^1^H NMR spectrum of OA-DA in DMSO-d_6_. **B**, High-resolution mass spectrum of OA-DA.

**Figure S7**. **A**, ^1^H NMR spectrum of LA-DA in DMSO-d_6_. **B**, High-resolution mass spectrum of LA-DA.

**Figure S8**. **A**, ^1^H NMR spectrum of LNA-DA in DMSO-d_6_. **B**, High-resolution mass spectrum of LNA-DA.

**Figure S9**. **Dose-dependent binding of OA-DA to HA-PBA.** The binding affinity of OA-DA was assessed by plotting the relative fluorescence intensity (I/I₀) against OA-DA concentration. Data were fitted to a binding curve, yielding an association constant (Kₐ) of 0.713 μM⁻¹, indicating a moderate affinity between OA-DA and HA-PBA.

**Figure S10**. FT-IR spectrums of HA, HA-PBA and HA-OA.

**Figure S11**. **A**) Size distribution and **B**) zeta potentials of lipidated catechols nano-assemblies and uhNAs. Insert, photograph of lipidated catechols nano-assemblies and uhNAs solutions at a HA-equivalent concentration of 0.1 mg mL^-1^. Size distribution of HA-PBA was also included in (**A**).

**Figure S12**. Determination of the critical aggregation concentration (CAC) of **A**) uhNAs-OA, **B**) uhNAs-LA, **C**) uhNAs-LNA, and **D**) HA-PBA by concentration-dependent light-scattering intensity measurements (kcps).

**Figure S13**. ^1^H NMR spectrum of Cy3-DA in DMSO-d_6_.

**Figure S14**. Morphology change of uhNAs-OA under acidic and oxidative conditions, as examined by TEM observation.

**Figure S15**. Stability of uhNAs-OA under acidic conditions (pH 6.0) or in the presence of 200 μM H_2_O_2_. Particle sizes and polydisperse index (PDI) were monitored *via* DLS analysis for 24 h.

**Figure S16**. Biodistribution of Cy5.5-labeled formulations in healthy and DSS-induced colitic mice. **A**) Representative fluorescence images of excised colons and major organs at 6 h. **B**) Quantitative analysis of the Cy5.5 signal in excised colons and major organs at 6 h.

**Figure S17**. Disease activity index in each treatment group. Mice were treated as indicated in Figure 3A. n = 7 biologically independent samples.

**Figure S18**. Diarrhea scores in each treatment group on day 5. Mice were treated as indicated in Figure 3A. n = 7 biologically independent samples.

**Figure S19**. Immunofluorescence staining of colons indicated the attenuated NF-κB signaling in uhNAs-OA-treated M1-like macrophages. Scale bar, 20 µm. Mice were treated as indicated in Figure 3A. n = 7 biologically independent samples.

**Figure S20**. *In vivo* blockade of CD44 attenuated the therapeutic efficacy of uhNAs-OA in DSS-induced acute colitis. **A**) Treatment schedule. C57BL/6 mice given 2.5% DSS were administered by daily oral gavage of saline or 30 mg kg^-1^ of uhNAs-OA. Anti-CD44 antibody (clone KM81, Cedarlane, 20 µg/mouse) was intravenously administered 2 h prior to oral gavage of uhNAs-OA. On day 8, mice were sacrificed, colons were harvested. Healthy mice were included as a control. **B**) Body weights of mice following treatments. Colon images (**C**) and corresponding colon length (**D**) in each treatment group. Levels of IL-6 (**E**) and TNF-α (**F**) in colonic tissue determined by ELISA on day 8. n = 6 biologically independent samples. Statistical significance was examined by one-way ANOVA with Tukey’s multiple comparisons test. Continuous variables with a skewed distribution, such as IL-6 and TNF-α, were log10-transformed before analysis to approximate normality and stabilize variance.

**Figure S21**. Excised spleens were photographed (**A**) and weighed (**B**). Mice were treated as indicated in Figure 3F. n = 7 biologically independent samples.

**Figure S22**. Histological analysis of colonic damage from each treatment group. Scale bar, 100 µm. Mice were treated as indicated in Figure 3F. n = 7 biologically independent samples.

**Figure S23**. DHE staining of ROS in colonic tissues. Representative images **A**) and quantitative analysis **B**) of ROS levels in each treatment group based on five confocal images per mouse. Data were presented as the mean ± SD, n = 3 biologically independent animals. Scale bar: 100 µm.

**Figure S24**. Gene set enrichment analysis (GSEA) showing the reduced inflammation responses in BMDMs following uhNAs-OA treatment.

**Figure S25**. KEGG analysis of DEGs in OA-DA- *vs* uhNAs-OA-treated group identified the ECM-receptor interaction signaling which associated with the phenotypic shift from M1 to M2.

**Figure S26**. CLSM images identified the impaired nuclear translocation of NF-κB-p65 following uhNAs-OA exposure and IFN-γ and LPS stimulation in Raw264.7 cells. NF-κB-p65: antibody against NF-κB-p65 (red), nuclei: DAPI (blue).

**Figure S27**. KEGG analysis of DEGs in HA- *vs* HA-PBA-treated group showing the enrichment in Chemical carcinogenesis-reactive oxygen species signaling, corroborating phenylboronic acid-mediated ROS scavenging.

**Figure S28**. Spleen weights in each treatment group. Mice were treated as indicated in Figure 5A. On day 9, mice were sacrificed, and colons and spleens were harvested. n = 7 biologically independent samples.

**Figure S29**. Flow cytometry gating strategy for macrophages.

**Figure S30**. Flow cytometry gating strategy for Tregs.

**Figure S31**. Flow cytometry gating strategy for CD8^+^ T cells.

**Figure S32**. Representative flow cytometric plots of CD8^+^ T cells population (gated as CD45^+^CD8^+^ T cells) in colons. Mice were treated as indicated in Figure 5A. On day 9, mice were sacrificed, colons were collected for immune analysis.

**Figure S33**. Flow cytometry gating strategy for neutrophils.

**Figure S34**. Flow cytometry gating strategy for CD45^+^ cells in colons. Mice were treated as indicated in Figure 5A. On day 9, mice were sacrificed, colons were collected for cell sorting and sc-RNA sequencing.

**Figure S35**. Dot plot showing the expression of canonical marker genes for each subset. Color indicates the average expression of marker genes, and dot size indicates the percentage of cells expressing these genes.

**Figure S36**. KEGG enrichment analysis of DEGs in macrophages (**A**) and monocytes (**B**) from uhNAs-OA *vs* saline control.

**Figure S37**. Dot plot for the expression of marker genes in macrophage subsets. Color indicates the average expression of marker genes, and dot size indicates the percentage of cells expressing these genes.

**Figure S38**. Volcano plot showing the DEGs in the macrophage compartment of uhNAs-OA *vs* saline control-treated colons. Each dot represents one gene. The representative differential genes linked to the inflammatory response pathway are labeled.

**Figure S39**. Dot plot for the expression of marker genes in T-cell subsets. Color indicates the average expression of marker genes, and dot size indicates the percentage of cells expressing these genes.

**Figure S40**. GO enrichment analysis of downregulated DEGs in T cells from uhNAs-OA *vs* saline control, highlighting specific pathway related to inflammation.

**Figure S41**. KEGG enrichment analysis of downregulated DEGs (excluding S100a8 and S100a9) in B cells from uhNAs–OA vs saline control, highlighting specific pathway related to inflammation.

**Figure S42**. Representative flow cytometric plots of CD86^+^ M1-, CD206^+^ M2-like macrophages in colons. Mice were treated as indicated in Figure 7A. On days 9 and 16, mice were sacrificed, colons were collected for immune analysis.

**Figure S43**. Representative flow cytometric plots of Tregs in colons. Mice were treated as indicated in Figure 7A. On days 9 and 16, mice were sacrificed, colons were collected for immune analysis.

**Figure S44**. Representative flow cytometric plots of CD8^+^ T cells in colons. Mice were treated as indicated in Figure 7A. On days 9 and 16, mice were sacrificed, colons were collected for immune analysis.

**Figure S45**. H&E staining of major organs, including the heart, lung, spleen, and kidneys of mice. The scale bar = 200 µm. Mice were treated as indicated in Figure 8A. On day 27, mice were sacrificed, major organs were collected for histological analysis.

**Figure S46**. t-SNE plots showing the expression level of *Klf2* and *S1pr1* in T-cell subsets. Black dotted regions indicate the Tregs and naïve T cells.

**Reference**

1.	Lee Y, Sugihara K, Gillilland MG, Jon S, Kamada N, Moon JJ. Hyaluronic acid–bilirubin nanomedicine for targeted modulation of dysregulated intestinal barrier, microbiome and immune responses in colitis. *Nature Materials* **19**, 118-126 (2020).

2.	Cao F*, et al.* Artificial-enzymes-armed Bifidobacterium longum probiotics for alleviating intestinal inflammation and microbiota dysbiosis. *Nature Nanotechnology* **18**, 617-627 (2023).

3.	Wang MA-O*, et al.* Precisely Inhibiting Excessive Intestinal Epithelial Cell Apoptosis to Efficiently Treat Inflammatory Bowel Disease with Oral Pifithrin-α Embedded Nanomedicine (OPEN). *Advanced Materials* **35**, e2309370 (2023).
